# Predicting therapeutic clinical trial enrollment for adult patients with low- and high-grade glioma using supervised machine learning

**DOI:** 10.1126/sciadv.adt5708

**Published:** 2025-06-04

**Authors:** Mulki Mehari, Gayathri Warrier, Abraham Dada, Aymen Kabir, Aden P. Haskell-Mendoza, Arushi Tripathy, Rohan Jha, Edwin Nieblas-Bedolla, Joshua D. Jackson, Ariel T. Gonzalez, Ellery H. Reason, Ann Marie Flusche, Sheantel Reihl, Tara Dalton, Mikias Negussie, Cesar Nava Gonzales, Vardhaan S. Ambati, Annick Desjardins, Andy G. S. Daniel, Saritha Krishna, Susan Chang, Alyx Porter, Peter E. Fecci, Todd Hollon, Ugonma N. Chukwueke, Kimberly Badal, Annette M. Molinaro, Shawn L. Hervey-Jumper

**Affiliations:** ^1^ Department of Neurosurgery, University of California, San Francisco, San Francisco, CA 94143, USA.; ^2^ Department of Neurosurgery, Duke University Medical Center, Durham, NC 27710, USA.; ^3^ Department of Neurosurgery, Michigan Medicine, Ann Arbor, MI 48109, USA.; ^4^ Harvard Medical School, Boston, MA 02115, USA.; ^5^ Department of Neurosurgery, University of California, Los Angeles, Los Angeles, CA 90095, USA.; ^6^ Preston Robert Tisch Brain Tumor Center, Department of Neurosurgery, Duke University Medical Center, Durham, NC 27710, USA.; ^7^ Division of Neuro-Oncology and Weill Institute for Neurosciences, University of California, San Francisco, San Francisco, CA 94158, USA.; ^8^ Division of Neuro-Oncology, Department of Neurology, Mayo Clinic, Phoenix, AZ 85054, USA.; ^9^ Machine Learning in Neurosurgery Laboratory, Department of Neurosurgery, University of Michigan, Ann Arbor, MI 48109, USA.; ^10^ Center for Neuro-Oncology, Department of Medical Oncology, Dana-Farber Cancer Institute, Harvard Medical School, Boston, MA 02215, USA.; ^11^ Department of Surgery, Helen Diller Comprehensive Cancer Center, University of California, San Francisco, San Francisco, CA 94158, USA.; ^12^ Weill Institute for Neurosciences, University of California, San Francisco, San Francisco, CA 94158, USA.

## Abstract

Therapeutic clinical trial enrollment does not match glioma incidence across demographics. Traditional statistical methods have identified independent predictors of trial enrollment; however, our understanding of the interactions between these factors remains limited. To test the interactive effects of demographic, socioeconomic, and oncologic variables on trial enrollment, we designed boosted neural networks (BNNs) for all glioma patients (*n* = 1042), women (*n* = 445, 42.7%), and minorities (*n* = 151, 14.5%) and externally validated these models [whole cohort, *n* = 230; women, *n* = 89 (38.7%); minority, *n* = 66 (28.7%)]. For the whole-cohort BNN, the most influential variables on enrollment were oncologic variables, including KPS [total effect (TE), 0.327], chemotherapy (TE, 0.326), tumor location (TE, 0.322), and seizures (TE, 0.239). The women-only BNN exhibited a similar trend. Conversely, for the minority-only BNN, socioeconomic variables [insurance status (TE, 0.213), occupation classification (TE, 0.204), and employment status (TE, 0.150)] were most influential. These results may help prioritize patient-specific initiatives to increase accrual.

## Teaser

Supervised machine learning models were designed and externally validated to predict glioma therapeutic clinical trial enrollment.

## INTRODUCTION

Clinical trials offer high-quality medical care and help preserve hope for patients with cancer (*1*, *2*). However, national enrollment rates have yet to match disease incidence and mortality across demographics (*3*–*5*). Previous studies have used traditional statistical methods to identify independent factors associated with clinical trial enrollment (*6*–*8*); however, our understanding of the nuanced interactions between these factors has remained limited. Harnessing machine learning techniques may serve as an innovative approach to assess the influence of prognostic variables and their complex relationships on trial accrual, improving our understanding of how we can overcome enrollment obstacles for select groups of patients (*9*–*11*).

The National Institutes of Health Revitalization Act of 1993 established disease-specific accrual benchmarks to increase representation in clinical trials. Despite these benchmarks, recent inquiry demonstrates alarming incidence-specific and mortality-specific under-accrual of women and minority patients with low- and high-grade glioma (*3*). Specifically, women represent 44.1% of incident cases and 44.2% of disease mortality, but only 37.7% of trial accrual (*3*). Similarly, minority patients represent 16.9% of incident cases and 20.1% of disease mortality, but only 5.9% of trial accrual (*3*). Additionally, many of the landmark clinical research studies supporting modern-day cancer-directed therapies for patients with diffuse gliomas are built on datasets with suboptimal representation of women and minorities (*12*, *13*). Therefore, increasing the enrollment of women and minority patients in glioma therapeutic clinical trials is paramount for ensuring generalizability of clinical research.

Recent investigations have demonstrated the potential of supervised machine learning to improve various aspects of clinical trial design (*14*, *15*), including operational efficiency (*16*), patient eligibility screening (*17*–*19*), clinical trial matching (*20*), electronic health record data extraction (*21*, *22*), and dynamic adjustment of recruitment sites and duration (*23*, *24*). However, these studies have not focused on increasing equitable clinical trial enrollment for patients with brain cancer. Thus, this multicenter study strives to close this critical gap in the literature. The primary aim is to design and externally validate machine learning models to test the interactive effects of demographic, socioeconomic, and oncologic variables on clinical trial enrollment for the general glioma patient population, women, and minorities. The secondary aim is to evaluate factors influencing trial screening and assess unique enrollment drivers across subgroups.

## RESULTS

Patient characteristics for the development cohort [University of California, San Francisco (UCSF)] and the external validation cohort (Duke University, University of Michigan, and Dana Farber Cancer Institute) are presented in Table 1. The development cohort included 1042 patients, 445 of whom were women (42.7%) and 151 of whom were minorities (14.5%). The validation cohort included 230 patients, 89 of whom were women (38.7%) and 66 of whom were minorities (28.7%). The development and validation cohorts did not differ significantly by sex, but the validation cohort had a significantly higher percentage of Black/African American patients (11.7% versus 1.6%) and Hispanic/Latino patients (8.7% versus 5.5%) compared to the development cohort. The percentage of minority patients in the development cohort matched the percentage of adult glioma patients who are minorities in the United States (expected, 16.9%; and observed, 14.5%), but several demographics differed (American Indian/Alaska Native: expected, 0.5%; and observed, 0.4%; Asian: expected, 1.9%; and observed, 4.6%; Hispanic/Latino: expected, 8.6%; and observed, 5.5%; Black/African American: expected, 6.0%; and observed, 1.6%) (*3*). The overall percentage of minority patients in the validation cohort was greater than expected based on glioma incidence rates (expected, 16.9%; and observed, 28.7%) but approximated a midpoint between the percentage of adult minority glioma patients and minority individuals in the United States (~42.6%) (*3*, *25*). Demographics of the validation cohort matched or exceeded expected rates (American Indian/Alaska Native: expected, 0.5%; and observed, 0.9%; Asian: expected, 1.9%; and observed, 5.2%; Hispanic/Latino: expected, 8.6%; and observed, 8.7%; Black/African American: expected, 6.0%; and observed, 11.7%) (*3*). For the development cohort, 995 patients (95.5%) preferred English for medical communication. Six hundred forty-three patients (62.0%) had private insurance, 83 patients (8.0%) had Medi-Cal/Medicaid, and 182 patients (17.5%) had Medicare. Six hundred fifty-eight patients (63.2%) had World Health Organization (WHO) grade 4 gliomas. Additional details on the characteristics of the Duke University, University of Michigan, and Dana Farber Cancer Institute subsets of the external validation cohort are provided in table S1.

**Table 1.** Demographic, socioeconomic, and oncologic characteristics of the UCSF development cohort, by therapeutic clinical trial enrollment, and the external validation cohort. ISCO, International Standard Classification of Occupations; IQR, interquartile range; UCSF, University of California, San Francisco; WHO, World Health Organization; KPS, Karnofsky Performance Scale.

	UCSF trial participants (*n* = 350)	UCSF non-trial participants (*n* = 692)	*P* value	UCSF development cohort (*n* = 1042)	External validation cohort (*n* = 230)	*P* value
**Mean age (±SD)** ^*^	50.7 ± 14.1	51.2 ± 15.3	0.624	51.0 ± 14.9	52.6 ± 15.9	0.170
**Sex**			0.468			
Female	144 (41.0%)	301 (43.5%)		445 (42.7%)	89 (38.7%)	0.265
Male	206 (58.9%)	391 (56.5%)		597 (57.3%)	141 (61.3%)	
**Race**			0.310			<0.0001
White	316 (90.3%)	596 (86.1%)		912 (87.5%)	170 (73.9%)	
Native Hawaiian and Pacific Islander	5 (1.4%)	13 (1.9%)			0 (0.0%)	
Asian	16 (4.6%)	32 (4.6%)		66 (6.3%)	12 (5.2%)	
Black/African American	4 (1.1%)	13 (1.9%)		17 (1.6%)	27 (11.7%)	
American Indian/Alaska Native	1 (0.3%)	3 (0.4%)		4 (0.4%)	2 (0.9%)	
Other	6 (1.7%)	23 (3.3%)		29 (2.8%)	14 (6.1%)	
Unknown	2 (0.6%)	12 (1.7%)		14 (1.3%)	5 (2.2%)	
**Ethnicity**			0.112			0.146
Hispanic/Latino	14 (4.0%)	43 (6.2%)		57 (5.5%)	20 (8.7%)	
Not Hispanic/Latino	332 (94.9%)	632 (91.3%)		964 (92.5%)	204 (88.7%)	
Unknown	4 (1.1%)	17 (2.5%)		21 (2.0%)	6 (2.6%)	
**Minority status**			0.036			<0.0001
Minority	41 (11.7%)	110 (15.9%)		151 (14.5%)	66 (28.7%)	
Nonminority	305 (87.1%)	563 (81.4%)		868 (83.3%)	158 (68.7%)	
Unknown	4 (1.1%)	19 (2.8%)		23 (2.2%)	6 (2.6%)	
**Preferred language**			0.015			
English	343 (98.0%)	652 (94.2%)		995 (95.5%)		
Not English	5 (1.4%)	35 (5.1%)		41 (3.8%)		
Unknown	2 (0.6%)	5 (0.7%)		7 (0.7%)		
**Interpreter used**			0.179			
Yes	6 (1.7%)	24 (3.5%)		30 (2.9%)		
No	342 (97.7%)	660 (95.4%)		1002 (96.2%)		
Unknown	2 (0.6%)	8 (1.2%)		10 (1.0%)		
**Insurance status**			0.003			<0.0001
Private	242 (69.1%)	401 (58.0%)		643 (61.7%)	103 (44.8%)	
Medi-Cal/Medicaid	19 (5.4%)	64 (9.3%)		83 (8.0%)	22 (9.6%)	
Medicare	44 (12.6%)	138 (19.9%)		182 (17.5%)	73 (31.7%)	
Unknown	41 (11.7%)	79 (11.4%)		120 (11.5%)	29 (12.6%)	
Self-pay	4 (1.1%)	10 (1.5%)		14 (1.3%)	3 (1.3%)	
**Employment status**			0.030			<0.0001
Employed	174 (49.7%)	305 (44.1%)		479 (46.0%)	136 (59.1%)	
Unemployed	88 (25.1%)	188 (27.2%)		276 (26.5%)	24 (10.4%)	
Retired	82 (23.4%)	163 (23.6%)		245 (23.5%)	55 (23.9%)	
Unknown	6 (1.7%)	36 (5.2%)		42 (4.0%)	15 (6.5%)	
**Occupational status - ISCO code**			0.505			
1 - Managers	18 (5.1%)	35 (5.1%)		53 (5.1%)		
2 - Professionals	57 (16.3%)	75 (10.8%)		132 (12.7%)		
3 - Technicians and associate professionals	18 (5.1%)	27 (3.9%)		45 (4.3%)		
4 - Clerical support workers	5 (1.4%)	9 (1.3%)		14 (1.3%)		
5 - Service and sales workers	11 (3.1%)	25 (3.6%)		36 (3.5%)		
6 - Skilled agricultural, forestry, and fishery workers	3 (0.9%)	6 (0.9%)		9 (0.9%)		
7 - Craft and related trades worker	2 (0.6%)	9 (1.3%)		11 (1.1%)		
8 - Plant and machine operators, and assemblers	0 (0.0%)	2 (0.3%)		2 (0.2%)		
9 - Elementary occupations	1 (0.3%)	4 (0.6%)		5 (0.5%)		
0 - Armed forces occupations	1 (0.3%)	5 (0.7%)		6 (0.6%)		
Self-employed	9 (2.6%)	17 (2.5%)		26 (2.5%)		
On disability	14 (4.0%)	20 (2.9%)		34 (3.3%)		
Student	1 (0.3%)	1 (0.1%)		2 (0.2%)		
Unknown	210 (60.0%)	457 (66.0%)		667 (64.0%)		
**Median household income (IQR)** ^**†**^	$101,705 (73682.25–138,861.5)	$87,273 (66,085.75–117,614)	<0.0001	$92,614.5 (67,862–128,383)	$68,908 (56,262–93,022)	<0.0001
**Median percentage below poverty level (IQR)** ^**†**^	7.6 (5–11.8)	9 (6.2–13.725)	0.0002	8.5 (5.7–13)	10.1 (6.0–15.0)	0.003
**Marital status**			0.480			
Married/registered domestic	256 (72.9%)	479 (69.3%)		735 (70.5%)		
Partner/significant other						
Divorced/separated	21 (6.0%)	40 (5.8%)		61 (5.9%)		
Widowed	9 (2.6%)	25 (3.6%)		34 (3.3%)		
Single	62 (17.7%)	144 (20.8%)		206 (19.8%)		
Unknown	3 (0.9%)	3 (0.4%)		6 (0.6%)		
**Location**			0.035			
In-state	280 (80.0%)	509 (73.6%)		789 (75.7%)		
Out-of-state	68 (19.4%)	170 (24.6%)		238 (22.8%)		
International	2 (0.6%)	13 (1.9%)		15 (1.4%)		
**Median distance from hospital in miles (IQR)** ^**†**^	54.95 (23.35–112.75)	88.1 (31.6–190)	0.0005	73.4 (28–169)	61.3 (27.6–199.6)	0.010
**Tumor type**			0.124			
WHO grade 2-3 astrocytoma	57 (16.3%)	142 (20.5%)		199 (19.1%)		
WHO grade 2-3 oligodendroglioma	55 (15.71%)	130 (18.8%)		185 (17.8%)		
WHO grade 4 astrocytoma	17 (4.9%)	25 (3.6%)		42 (4.0%)		
WHO grade 4 glioblastoma	221 (63.1%)	395 (57.1%)		616 (59.1%)		
**WHO grade**			0.021			<0.0001
WHO grade 2-3	112 (32.0%)	272 (39.3%)		384 (36.9%)	46 (20.0%)	
WHO grade 4	238 (68.0%)	420 (60.7%)		658 (63.2%)	184 (80.0%)	
**Tumor location: hemisphere**			0.0004			
Left	190 (54.3%)	341 (49.3%)		531 (51.0%)		
Right	157 (44.9%)	318 (45.0%)		475 (45.6%)		
Bilateral	3 (0.9%)	2 (0.3%)		5 (0.5%)		
Unknown	0 (0.0%)	31 (4.5%)		31 (3.0%)		
**Tumor location: lobe**			0.0006			<0.0001
Brainstem, insular, basal ganglia, or thalamus	35 (10.0%)	52 (7.5%)		87 (8.4%)	10 (4.4%)	
Frontal	131 (37.4%)	280 (40.5%)		411 (39.4%)	88 (38.3%)	
Multifocal	39 (11.1%)	53 (7.7%)		92 (8.8%)	2 (0.87%)	
Occipital	9 (2.6%)	15 (2.2%)		24 (2.3%)	14 (6.1%)	
Parietal	61 (17.4%)	111 (16.0%)		172 (16.5%)	31 (13.4%)	
Temporal	74 (21.1%)	149 (21.5%)		223 (21.4%)	82 (35.7%)	
Cerebellum	0 (0.0%)	1 (0.1)%		1 (0.1%)	3 (1.3%)	
Unknown	1 (0.3%)	31 (4.5%)		32 (3.1%)	0 (0.0%)	
**Seizure**			0.0006			<0.0001
Yes	234 (66.9%)	377 (54.5%)		611 (58.6%)	38 (20.9%)	
No	109 (31.1%)	297 (42.9%)		406 (39.0%)	54 (23.5)	
Unknown	7 (2.0%)	18 (2.6%)		25 (2.4%)	128 (55.7%)	
**Preoperative tumor volume in milliliters**			0.330			
<25	66 (18.9%)	156 (22.5%)		222 (21.3%)		
25–49	93 (26.6%)	152 (22.0%)		245 (23.5%)		
50–99	102 (29.1%)	191 (27.6%)		293 (28.1%)		
100–400	88 (25.1%)	192 (27.8%)		280 (27.9%)		
Unknown	1 (0.3%)	1 (0.1%)		2 (0.2%)		
**Volumetric extent of resection (%)**			0.738			
<80	225 (64.3%)	434 (62.75)		659 (63.2%)		
≥80	124 (35.4%)	254 (36.7%)		378 (36.3%)		
Unknown	1 (0.3%)	4 (0.6%)		5 (0.5%)		
**Chemotherapy**			<0.0001			<0.0001
Yes	307 (87.7%)	441 (63.7%)		748 (71.8%)	209 (90.9%)	
No	38 (10.9%)	223 (32.2%)		261 (25.1%)	15 (6.5%)	
Unknown	5 (1.4%)	28 (4.1%)		33 (3.2%)	6 (2.6%)	
**Radiation**			<0.0001			
Yes	279 (80.0%)	457 (66.0%)		736 (70.6%)		
No	60 (17.1%)	189 (27.3%)		249 (23.9%)		
Unknown	11 (3.1%)	46 (6.7%)		57 (5.5%)		
**KPS**			<0.0001			<0.0001
<80	31 (8.9%)	123 (17.8%)		154 (14.8%)	57 (24.8%)	
≥80	264 (75.4%)	342 (49.4%)		606 (38.2%)	170 (73.9%)	
Unknown	55 (15.7%)	277 (32.8%)		282 (27.1%)	3 (1.3%)	

*Difference calculated using a two-tailed *t* test.

†Difference calculated using a Wilcoxon rank sum test.

Of the 1042 patients with a median follow-up time of 28.8 months (interquartile range, 12.1 to 106.1), 628 (60.3%) were screened for, and 350 (33.6%) enrolled in a glioma therapeutic clinical trial (table S1). One hundred forty-four of the 445 women (32.4%) and 41 of the 151 minority patients (27.2%) enrolled in a trial (tables S2 and S3). Clinical trial screening rates (validation versus development, 80.9% versus 60.3%) and enrollment rates (validation versus development, 58.7% versus 33.6%) were higher for the validation cohort than for the development cohort. Patient characteristics for the women and minority subsets of the development and validation cohorts are presented in tables S2.

### Machine learning prediction of clinical trial enrollment and screening

#### Model screening with internal validation

On internal cross-validation, the boosted neural network (BNN) model achieved the best predictive performance for enrollment [mean area under the curve (AUC) across 10 folds, 0.7840; accuracy, 0.7249; misclassification rate (MR), 0.2743; Mathew correlation coefficient (MCC), 0.3849] and was thus selected for external validation (table S4).

#### Model performance on validation cohort for trial screening

Development models were computed using patients at UCSF and validated against glioma patients treated at Duke University, University of Michigan, and Dana-Farber Cancer Institute. The whole-cohort BNN model achieved good predictive performance for trial screening in both the development [AUC, 0.8677; accuracy, 79.9%; MR, 0.201; MCC, 0.575] and validation (AUC, 0.8372; accuracy, 84.4%; MR, 0.157; MCC, 0.4274) cohorts (fig. S1). The women-only BNN model (development: AUC, 0.8915; accuracy, 79.8%; MR, 0.202; MCC, 0.569; validation: AUC, 0.8575; accuracy, 83.2%; MR, 0.169; MCC, 0.4971) and the minority-only BNN model (development: AUC, 0.930; accuracy, 87.4%; MR, 0.126; MCC, 0.734; validation: AUC, 0.8202; accuracy, 83.6%; MR, 0.164; MCC, 0.603) also accurately predicted trial screening (fig. S1).

For all cohorts, the most influential variables predicting trial screening were oncologic variables, including treatment with chemotherapy [whole cohort: total effect (TE), 0.344; women: TE, 0.354; and minority: TE, 0.140], Karnofsky Performance Scale (KPS) (whole cohort: TE, 0.308; women: TE, 0.295; and minority: TE, 0.216), and tumor location (whole cohort: TE, 0.219; women: TE, 0.289; and minority: TE, 0.198), followed by a blend of socioeconomic, demographic, and other oncologic variables (fig. S1).

#### Model performance on validation cohort for trial enrollment

**Fig. 1.**
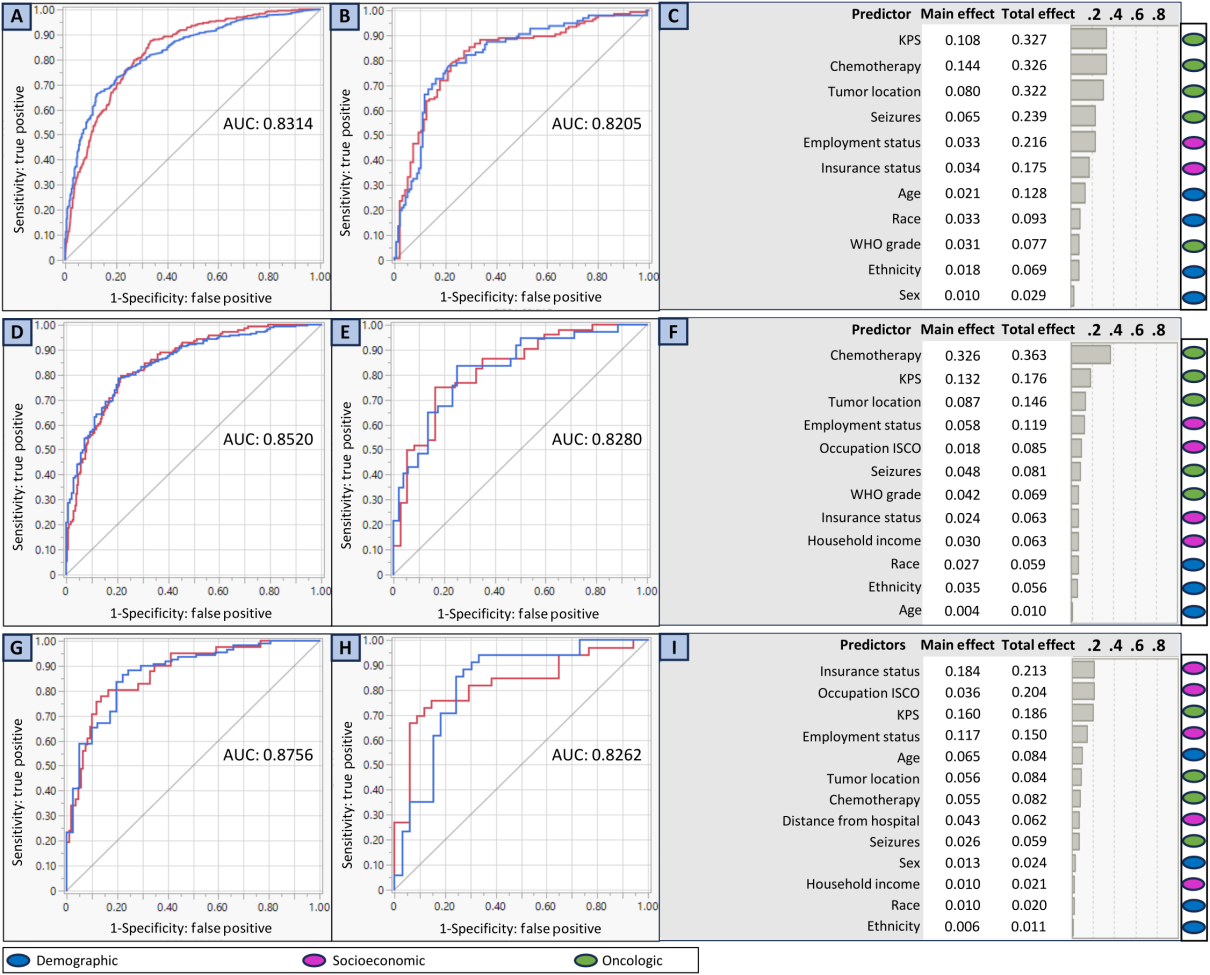
ROC curves and ranked variables of importance for BNN models for trial enrollment. The figure depicts the receiver operator characteristic (ROC) curves and variables of importance for predicting therapeutic clinical trial enrollment using BNN models for the whole, women-only, and minority-only cohorts. In all ROC curves, the red lines represent clinical trial enrollment, while the blue lines represent lack of enrollment. The AUC values for each ROC curve are listed. The main and total effects for each variable are listed in variables of importance output for each model. Adjacent to the variables of importance outputs are color-coded heatmaps indicating whether the ranked factors are demographic, socioeconomic, or oncologic variables. (**A**) The ROC curve for the whole development cohort. (**B**) The ROC curve for the whole validation cohort. (**C**) The variables of importance for the whole-cohort model in descending order of importance. (**D**) The ROC curve for the women development cohort. (**E**) The ROC curve for the women validation cohort. (**F**) The ranked variables of importance for the women cohort model in descending order of importance. (**G**) The ROC curve for the minority development cohort. (**H**) The ROC curve for the minority validation cohort. (**I**) The ranked variables of importance for the minority cohort model in descending order of importance.

The whole-cohort BNN model achieved good predictive performance in both the development (AUC, 0.8314; accuracy, 76.6%; MR, 0.234; MCC, 0.461) and validation (AUC, 0.8205; accuracy, 76.1%; MR, 0.239; MCC, 0.523) cohorts (Fig. 1). The women-only BNN model (development: AUC, 0.8520; accuracy, 78.7%; MR, 0.213; MCC, 0.502; validation: AUC, 0.8280; accuracy, 71.3%; MR, 0.281; MCC, 0.469) and the minority-only BNN model (development: AUC, 0.8756; accuracy, 84.1%; MR, 0.159; MCC, 0.598; validation: AUC, 0.8262; accuracy, 77.6%; MR, 0.224; MCC, 0.583) also accurately predicted enrollment (Fig. 1).

The most influential variables in predicting trial enrollment varied by cohort (Fig. 1). For the whole-cohort BNN model, oncologic variables, including KPS (TE, 0.327), chemotherapy (TE, 0.326), tumor location (TE, 0.322), and seizures (TE, 0.239), were most influential on enrollment, followed by socioeconomic [employment status (TE, 0.216) and insurance status (TE, 0.175)] and demographic [age (TE, 0.128) and race (TE, 0.093)] variables (Fig. 1C). Similarly, for the women-only BNN model, oncologic variables [chemotherapy (TE, 0.363), KPS (TE, 0.176), and tumor location (TE, 0.146)] were most influential; however, a blend of socioeconomic [employment status (TE, 0.119), occupation classification (TE, 0.085), insurance status (TE, 0.063), and household income (TE, 0.063)], and other oncologic variables [seizures (TE, 0.081) and WHO grade (TE, 0.069)] were also highly influential (Fig. 1F).

In contrast, for the minority-only BNN model, the most influential predictors were socioeconomic variables [insurance status (TE, 0.213), occupation classification (TE, 0.204), and employment status (TE, 0.150)], followed by a mix of oncologic [KPS (TE, 0.186), tumor location (TE, 0.0804), chemotherapy (TE, 0.082), and seizure (TE, 0.059)], demographic [age (TE, 0.084) and sex (TE, 0.024)], and other socioeconomic [distance from hospital (TE, 0.062) and household income (TE, 0.021)] variables (Fig. 1I).

Next, given slight differences in minority populations between institutions included in this study, we performed additional analysis on a combined cohort of minority-only patients treated at UCSF, Duke, Michigan, and Dana-Farber Cancer Institute. Similar to our main minority-only BNN model, the combined minority cohort BNN model prioritized socioeconomic variables [occupation classification (TE, 0.373), insurance status (TE, 0.335), and employment status (TE, 0.263) as the three variables most influential on enrollment] (fig. S1).

**Fig. 2.**
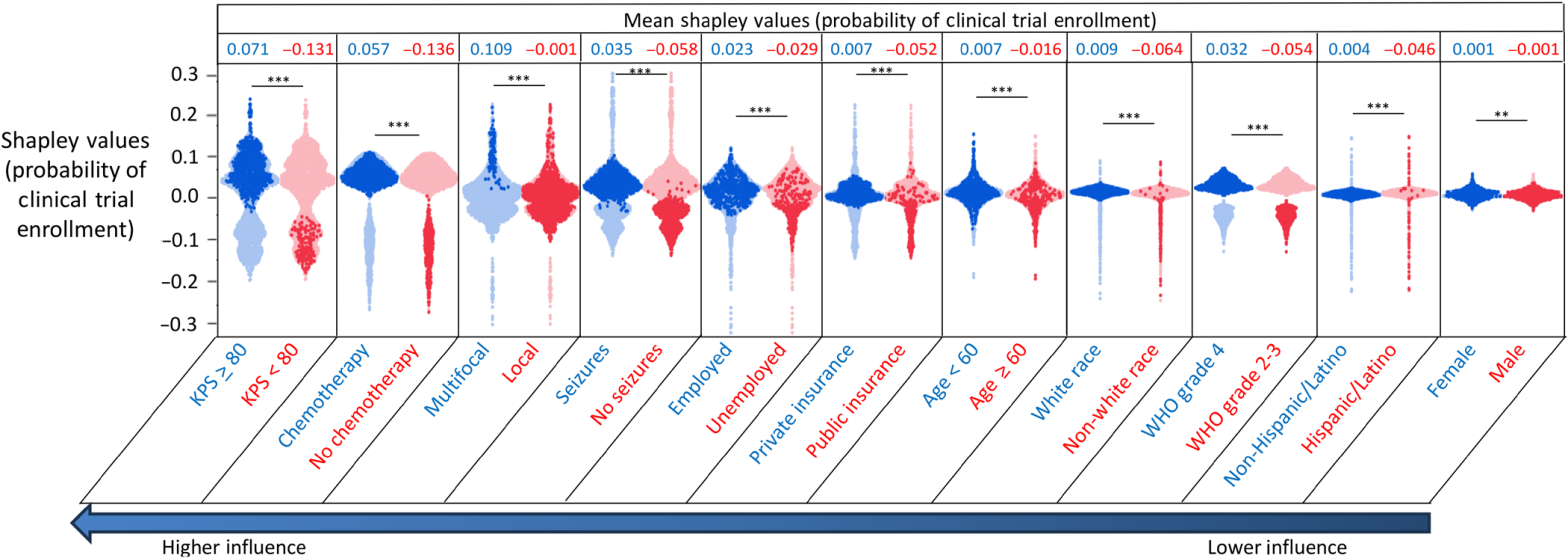
Shapley values for influential predictors for the whole-cohort BNN model. The figure shows the distribution of Shapley values for each influential predictor in the whole-cohort BNN model. Each dot corresponds to the Shapley value for each predictor for a single patient. Positive and negative Shapley values indicate increased and decreased probability of enrollment, respectively. Each predictor is stratified into two levels, with the characteristic associated with higher mean Shapley values color coded in dark blue and the characteristic with the lower mean Shapley values color coded in dark red. *t* tests were used to assess for differences in mean Shapley values for each predictor. Predictors are arranged from most influential to least influential (left to right) according to the variables of importance calculation for the predictive model. **P < 0.01; ***P < 0.0001.

Mean Shapley values were calculated to assess the directionality of each predictor, with positive and negative values indicating increased and decreased probability of enrollment, respectively. For the whole-cohort BNN model, mean Shapley values were significantly higher (*P* < 0.0001) for KPS ≥ 80 (versus <80, 0.071 versus −0.131), treatment with chemotherapy (versus no chemotherapy, 0.057 versus −0.136), multifocal tumor location (versus local, 0.109 versus −0.001), presenting seizures (versus no seizures, 0.035 versus −0.058), active employment (versus unemployment, 0.023 versus −0.029), private insurance (versus public insurance, 0.007 versus −0.052), age less than 60 years (versus >60, 0.007 versus −0.016), white race (versus non-white, 0.009 versus −0.064), WHO grade 4 (versus grade 2-3, 0.030 versus −0.054), and non-Hispanic ethnicity (versus Hispanic ethnicity, 0.004 versus −0.046) (Fig. 2). The mean Shapley value was significantly higher for female sex than male sex (0.001 versus −0.001, *P* < 0.0001), although the net difference in mean Shapley values was slight (0.002). The directionality and significance of these relationships were mainly maintained for the women and minority BNN models (figs. S2 to S4). Additionally, there were unique relationships for Shapley values for the women and minority-only BNN models. For both the women and minority BNN models, mean Shapley values were significantly higher for patients with median household income by zip code greater than $75,000 (women: versus less than $75,000, 0.019 versus −0.031, *P* < 0.0001; minority: versus less than $75,000, 0.003 versus −0.026, *P* < 0.0001) and patients with higher International Standard Classification of Occupations (ISCO) skill level occupations (women: ISCO skill level 3 to 4 versus ISCO skill level 1 to 2, 0.005 versus −0.032, *P* < 0.0001; minority: ISCO skill level 4 versus ISCO skill level 1 to 2, 0.018 versus −0.057, *P* < 0.0001). Last, for the minority BNN model, the mean Shapley value was significantly higher for travel distance less than 50 miles (0.054 versus −0.007, *P* < 0.0001).

### Factors associated with clinical trial enrollment on multivariate logistic regression

For the whole cohort, higher household income {odds ratio (OR) [95% confidence interval (CI)], 1.079 per $10,000 increase (1.029 to 1.132); *P* = 0.002}, higher WHO grade [4 versus 2-3; OR (95% CI), 1.609 (1.049 to 2.467); *P* = 0.029], seizure [OR (95% CI), 1.630 (1.181 to 2.249); *P* = 0.003], and higher KPS [≥80 versus <80; OR (95% CI), 2.802 (1.729 to 4.540); *P* < 0.0001] were associated with increased odds of enrollment. Non-English preferred language [OR (95% CI), 0.005 (0.0001 to 0.206); *P* = 0.005], lack of interpreter use [OR (95% CI), 0.011 (0.0003 to 0.426); *P* = 0.016], Medicare insurance [OR (95% CI), 0.497 (0.300 to 0.822); *P* = 0.007], and lack of chemotherapy [OR (95% CI), 0.248 (0.150 to 0.409); *P* < 0.0001] were associated with decreased odds of enrollment (Table 2).

**Table 2.** Univariate and multivariate logistic regression of therapeutic clinical trial enrollment for the whole cohort. OR, odds ratio; CI, confidence interval; ISCO, International Standard Classification of Occupations; UCSF, University of California, San Francisco; WHO, World Health Organization; KPS, Karnofsky Performance Scale; *undefined upper limit of confidence interval.

	Whole cohort			
	Univariate logistic regression		Multivariate logistic regression	
	**OR (95% CI)**	***P* value**	**OR (95% CI)**	***P* value**
**Age**	0.979 (0.898–1.067)	0.632		
**Sex**				
Female	0.910 (0.700–1.178)	0.470		
Male	Referent			
**Race**		0.255		
White	Referent			
American Indian/Alaska Native	0.629 (0.065–6.069)	0.688		
Asian	0.943 (0.510–1.745)	0.852		
Black/African American	0.580 (0.188–1.795)	0.345		
				
Native Hawaiian and Pacific Islander	0.725 (0.256–2.053)	0.545		
Other race(s)	0.492 (0.198–1.221)	0.126		
Unknown	0.314 (0.070–1.413)	0.131		
**Ethnicity**		0.095		
Hispanic/Latino	0.620 (0.334–1.149)	0.129		
Not Hispanic/Latino	Referent			
Unknown	0.448 (0.150–1.342)	0.151		
**Minority status**		0.032		
Minority	0.688 (0.468–1.011)	0.057		
Nonminority	Referent			
Unknown	0.389 (0.131–1.153)	0.088		
**Preferred language**		0.007		0.003
English	Referent		Referent	
Not English	0.272 (0.105–0.699)	0.007	0.005 (0.0001–0.206)	0.005
Unknown	0.760 (0.147–3.939)	0.744	0.326 (0.010–10.424)	0.526
**Interpreter used**		0.153		0.017
Yes	Referent		Referent	
No	2.070 (0.839–5.119)	0.110	0.011 (0.0003–0.426)	0.016
Unknown	1.000 (0.167–5.985)	1.000	0.526 (0.018–15.426)	0.709
**Insurance status**		0.002		0.014
Private	Referent		Referent	
Medi-Cal/Medicaid	0.492 (0.288–0.841)	0.001	0.576 (0.307–1.081)	0.086
Medicare	0.528 (0.363–0.769)	0.001	0.497 (0.300–0.822)	0.007
Unknown	0.860 (0.571–1.300)	0.470	1.291 (0.779–2.138)	0.322
Self-pay	0.663 (0.206–2.137)	0.491	1.111 (0.263–4.694)	0.886
**Employment status**		0.018		
Employed	Referent			
Unemployed	0.820 (0.599–1.123)	0.217		
Retired	0.882 (0.638–1.219)	0.447		
Unknown	0.292 (0.121–0.707)	0.006		
**Occupational status - ISCO code**		0.454		
1 - Managers	Referent			
2 - Professionals	1.478 (0.760–2.873)	0.2495		
3 - Technicians and associate professionals	1.296 (0.569–2.955)	0.5370		
4 - Clerical support workers	1.080 (0.315–3.704)	0.9023		
5 - Service and sales workers	0.856 (0.345–2.123)	0.7366		
6 - Skilled agricultural, forestry, and fishery workers	0.972 (0.217–4.348)	0.9706		
7 - Craft and related trades worker	0.432 (0.084–2.215)	0.314		
8 - Plant and machine operators, and assemblers	0.000 (0.000–*)	0.987		
9 - Elementary occupations	0.486 (0.051–4.676)	0.532		
0 - Armed forces occupations	0.389 (0.042–3.585)	0.405		
Other: self-employed	1.029 (0.383–2.765)	0.954		
On disability	1.361 (0.560–3.310)	0.497		
Student	1.944 (0.115–32.933)	0.645		
Unknown	0.894 (0.495–1.614)	0.709		
**Household income (per $10,000 increase)**	1.071 (1.041–1.103)	<0.0001	1.079 (1.029–1.132)	0.002
**Percentage below poverty level**	0.968 (0.946–0.990)	0.0036		
**Marital status**		0.4777		
Married/registered domestic	Referent			
Partner/significant other				
Divorced/separated	0.982 (0.567–1.702)	0.949		
Widowed	0.674 (0.310–1.465)	0.319		
Single	0.787 (0.563–1.101)	0.162		
Unknown	1.871 (0.375–9.337)	0.445		
**Location**		0.027		
In-state	Referent			
Out-of-state	0.727 (0.530–0.998)	0.049		
International	0.280 (0.063–1.248)	0.095		
**Distance from UCSF if in state in miles**	0.975 (0.962–0.988)	0.0002		
**Tumor type**		0.122		
WHO grade 2-3 astrocytoma	Referent			
WHO grade 2-3 oligodendroglioma	1.054 (0.679–1.637)	0.815		
WHO grade 4 astrocytoma	1.694 (0.851–3.372)	0.134		
WHO grade 4 glioblastoma	1.394 (0.984–1.975)	0.0619		
**WHO grade**		0.0203		0.029
WHO grade 2-3	Referent		Referent	
WHO grade 4	1.376 (1.049–1.805)	0.0212	1.609 (1.049–2.467)	0.029
**Tumor location: hemisphere**		<0.0001		
Left	Referent			
Right	0.886 (0.683–1.150)	0.363		
Bilateral	2.692 (0.446–16.253)	0.280		
Unknown	0.000 (0.000–*)	0.986		
**Tumor location: lobe**		0.001		
Brainstem, insular, basal ganglia, or thalamus	1.439 (0.894–2.316)	0.134		
Frontal	Referent			
Multifocal	1.602 (1.006–2.551)	0.047		
Occipital	1.282 (0.547–3.007)	0.567		
Parietal	1.175 (0.807–1.709)	0.400		
Temporal	1.062 (0.750–1.503)	0.736		
Cerebellum	0.000 (0.000–*)	0.987		
Unknown	0.069 (0.009–0.511)	0.009		
**Seizure**		0.0006		0.011
Yes	1.691 (1.287–2.223)	0.0002	1.630 (1.181–2.249)	0.003
No	Referent		Referent	
Unknown	1.060 (0.431–2.607)	0.8996	1.445 (0.519–4.026)	0.481
**Preoperative tumor volume in milliliters**		0.3325		
<25	Referent			
25–49	1.446 (0.983–2.129)	0.061		
50–99	1.262 (0.868–1.837)	0.224		
100–400	1.083 (0.739–1.159)	0.682		
Unknown	2.364 (0.146–38.356)	0.545		
**Volumetric extent of resection (%)**		0.7234		
<80	1.062 (0.812–1.389)	0.6607		
≥80	Referent			
Unknown	0.512 (0.057–4.630)	0.5514		
**Chemotherapy**		<0.0001		<0.0001
Yes	Referent		Referent	
No	0.245 (0.168–0.356)	<0.0001	0.248 (0.150–0.409)	<0.0001
Unknown	0.257 (0.098–0.672)	0.006	0.346 (0.120–1.000)	0.050
**Radiation**		<0.0001		
Yes	Referent			
No	0.520 (0.375–0.721)	<0.0001		
Unknown	0.392 (0.200–0.769)	0.007		
**KPS**		<0.0001		<0.0001
<80	Referent		Referent	
≥80	3.063 (2.002–4.687)	<0.0001	2.802 (1.729–4.540)	<0.0001
Unknown	0.961 (0.588–1.572)	0.875	1.219 (0.678–2.192)	0.508

Among patients screened for a trial, discussing more trials during screening {OR (95% CI): 2 trials, [2.101 (1.336 to 3.302); *P* = 0.001]; 3 trials, [OR 2.201 (1.107 to 4.377); *P* = 0.025]; and 5+ trials, [5.073 (1.065 to 24.174); *P* = 0.042]} was associated with a dose-dependent increase in odds of enrollment (table S3). Fewer trials were discussed with women (versus men; means ± SD, 1.61 ± 0.96 versus 1.89 ± 1.16; *P* = 0.0001), minorities (versus non-minorities; means ± SD, 1.49 ± 0.63 versus 1.81 ± 1.14; *P* = 0.0002), and patients who preferred a non-English language (versus English; means ± SD, 1.25 ± 0.45 versus 1.78 ± 1.09; *P* = 0.002) (table S4). Higher household income, seizure, higher KPS, and treatment with chemotherapy were similarly associated with trial screening (table S5). No unique variables were associated with enrollment among only women or only minority patients (table S6).

## DISCUSSION

We designed and externally validated machine learning models to accurately predict therapeutic clinical trial enrollment for patients with low- and high-grade glioma. Our BNN models performed well across cohorts for trial enrollment and screening. For screening, oncologic variables were most influential across all cohorts. For enrollment, oncologic variables continued to be most influential for the whole-cohort and women models; however, for minority patients, socioeconomic variables were most influential. The directionality of influence of enrollment predictors was maintained across cohorts.

For the whole-cohort model, the oncologic variables of higher KPS, multifocal tumor, treatment with chemotherapy, and presenting seizures were most influential on enrollment and associated with a higher average probability of enrollment. The multivariate logistic regression analysis substantiated these relationships. Collectively, these findings suggest that higher symptom burden may motivate patients to enroll if their functional status meets inclusion criteria and they are willing to undergo cancer-directed treatments, such as chemotherapy. There may be a specific significance of tumor location influencing enrollment as patients with a tumor that is multifocal or difficult to resect meaningfully may derive limited potential benefits from standard treatment and instead pursue investigational therapies.

In multivariate logistic regression analysis, race and sex were not found to be independently associated with clinical trial enrollment. Further, multivariate regressions for the women and minority subgroups did not identify unique drivers of enrollment. However, the BNN models for all cohorts suggest that race, ethnicity, and sex are essential enrollment drivers. For example, non-white race and non-Hispanic ethnicity were associated with lower average probability of enrollment. Thus, in contrast to conventional statistical analysis, the BNN models can capture the interconnectedness between demographic and socioeconomic variables in influencing enrollment in a manner that parallels how such characteristics influence complex enrollment decisions for a unique patient (*9*). Therefore, this study can serve as a bridge to an era of precision clinical trial research in which machine learning techniques can map which factors most influence enrollment for individual patients to guide personalized recruitment interventions to increase accrual (*11*). On the other hand, our minority cohort model underscores the importance of addressing root causes of under-accrual in trials. For example, the influence of socioeconomic variables on enrollment was more pronounced for minority patients, with higher ISCO skill level occupations, active employment, and private insurance having a higher average probability of enrollment. These findings may reflect patient-specific differences in occupation, income, health literacy, and health insurance, which produce and exacerbate gaps in trial enrollment for minority patients (*26*, *27*). While we assessed factors influencing enrollment for minority glioma patients in aggregate, we did not explore how these factors may differ for specific racial and ethnic groups, representing opportunities for future research.

In contrast to national trends, baseline enrollment rates for men and women in our study were comparable. In the women-only cohort, non-white race was associated with lower probability of enrollment, underscoring how race and sex can intersect to influence accrual (*28*). Therefore, recruiting more women may fall short of achieving benchmark accrual of minority women. Unexpectedly, in our BNN model, the mean Shapley value for female sex was slightly higher than that for male sex (*3*). This counterintuitive finding could relate to a smaller sample size in this multi-institution study than national cohort studies or unique institutional factors. An alternative explanation is that prior studies focused on the under-accrual of women in glioma trials have not used statistical methods that fully account for covariates, such as differences in oncologic or socioeconomic factors (*3*). Our BNN model raises the possibility that, when accounting for these complex interactions, female sex may not be associated with lower probability of enrollment but potentially even a slight increase in enrollment probability; however, this hypothesis requires additional exploration including larger patient cohorts.

We found that oncologic variables were consistently most influential in predicting trial screening across all cohorts, suggesting that clinical status triggers clinicians to discuss trial options with patients. We also found that discussing more trials during screening was associated with progressively higher odds of enrollment. However, fewer trials were discussed with women, minorities, and patients who preferred a non-English language. This disparity may limit shared decision-making and relate to factors including baseline patient interest or socioeconomic obstacles. Further, patients underrepresented in clinical trials may be less likely to initiate discussions with their clinicians about clinical trial options due to differences in health literacy or medical mistrust (*29*, *30*). Therefore, encouraging clinicians to adopt a “just ask” policy for clinical trials discussion for all eligible patients, increasing provider awareness of available trials, and maximizing the number of trials discussed during screening may increase accrual. Further, among patients who preferred a non-English language, not using an interpreter was associated with an additional reduction in odds of enrollment. Therefore, it is imperative to not only democratize access to interpreter services but also prioritize their use during medically complex screening and enrollment discussions, even for patients with moderate English proficiency who otherwise might forego medical interpretation for other healthcare encounters (*31*, *32*).

Notably, non-oncological attributes are influential variables on trial enrollment (*33*). As a result, if our BNN model was used to target recruitment of patients with higher projected enrollment probability without efforts to address barriers to enrollment, then this could exacerbate the under-accrual of vulnerable groups of patients (*3*, *4*, *34*). This is particularly relevant given prior studies showing an association between trial enrollment and improved survival for patients with high-grade glioma (*35*). Ideally, future efforts will result in balanced datasets for algorithm training; however, in the path toward reaching this goal, researchers must identify and address model bias through bias-correcting techniques before deployment in the clinical setting (*36*, *37*).

There are several limitations of this study. First, while “black box” neural networks obscure relationships between variables and outcomes (*38*, *39*), we used Shapley values to help explain model decision-making (*40*). However, given that a few predictors were moderately correlated, our SHapley Additive exPlanations (SHAP) analysis could overestimate the individual contributions of correlated features. However, the risk of predictor misattribution is reduced because of the lack of highly correlated predictors. Second, the smaller sample size of minority patients may have prevented identification of unique drivers of enrollment on multivariate logistic regression. Furthermore, the sample size of minority patients in the development and validation cohorts creates the risk of overfitting our minority-only BNN model. Third, the racial and ethnic breakdowns of the development and validation cohorts may not perfectly reflect national demographics, which could lead to a different ranking of influential variables than found for centers in other regions of the United States. To overcome these limitations, we pooled the minority patients from the development and validation datasets to augment sample size and generate a more generalizable model, which prioritized similar model features. Fourth, because we relied on documentation in the electronic medical record to determine whether patients were screened for clinical trials, there is the possibility that some of these patient-clinician discussions occurred but were not documented. Fifth, our models were unable to capture the variance in the number of clinical trials available to each patient throughout the study period, which may have influenced recruitment rates. Sixth, enrollment outcomes may have been affected by patients lost to follow-up. Furthermore, certain patients in the development and training cohorts may have enrolled in a therapeutic clinical trial at an outside institution that our datasets could not capture due to nonintegrated electronic health records, leading to potential misclassification of a small number of patients. Last, zip codes were used as a proxy for income and poverty at the population level, which may not perfectly match the socioeconomic characteristics of individual patients.

To facilitate the evaluation of our BNN model’s predictive performance in additional settings and cohorts, we created GPREDICT (prediction of glioma patients’ enrollment in therapeutic clinical trials). This open-access tool estimates the probability that a given patient with a glioma will enroll in a therapeutic clinical trial based on patient, oncologic, and socioeconomic characteristics inputted by the user. GPREDICT also provides general recommendations for clinical trial screening discussions, identifies the patient factors that most influence estimated enrollment probability, and provides users with suggestions for targeted interventions to increase each patient’s accrual probability. The GPREDICT tool is available here: https://mmehari2025.github.io/glioma-trial-predictor/.

This study can serve as a bridge to an era of precision clinical trial enrollment research that harnesses machine learning techniques to optimize clinical trial participation for all patients with glioma. These findings can guide tailored efforts to promote equitable accrual across diverse patient populations.

## MATERIALS AND METHODS

### Study design

**Fig. 3.**
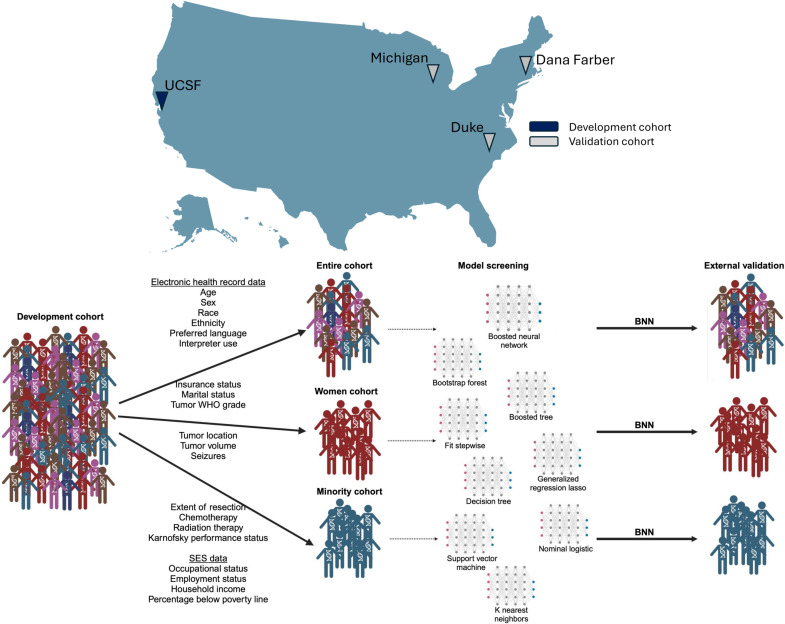
GPREDICT schematic for BNN development and validation for the whole cohort, women-only cohort, and minority-only cohort. The figure represents four academic medical centers, and the schematic depicts the sequence of dividing the whole cohort into the women-only and minority-only cohorts for model development, model screening with internal cross-validation, and external validation.

In this retrospective, multicenter cohort study, we trained machine learning algorithms to predict therapeutic clinical trial screening (secondary outcome) and enrollment (primary outcome). Separate models were generated for all patients, women, and minority patients in the development cohort and then independently validated in their respective external cohorts, as depicted in Fig. 3. Minority patients were defined as any patient with Black or African American, Asian, American Indian or Alaska Native, or Native Hawaiian or Pacific Islander race and/or Latino or Hispanic ethnicity according to the current United States Office of Management and Budget’s Race/Ethnicity Standards (*41*). This study was approved by the institutional review boards of the UCSF. Duke University, University of Michigan, and the Dana Farber Cancer Institute. Additional methods are included in Supplementary Text.

One thousand forty-two adult patients with low- and high-grade glioma who received care from the UCSF Brain Tumor Center between 1 January 1997 and 31 December 2017 were identified in the UCSF Cancer Registry. Twenty-four demographic, socioeconomic, oncologic, and therapeutic clinical trial screening and enrollment variables were extracted from the registry and electronic health record (Table 1). Therapeutic clinical trial screening was defined as a chart documented discussion about trial enrollment during consultation with neuro-oncology or neurosurgery. Clinical trial enrollment was defined as enrolling in a therapeutic phase I-IV clinical trial at UCSF. At UCSF, there were 238 therapeutic trials for glioma open at the UCSF Brain Tumor Center from January 1997 to December 2017. The total enrollment during this time period was 2661 patients, equating to an average annual enrollment of 126 patients per year.

External validation was performed on a combined cohort of adult glioma patients from three external institutions: Duke University, University of Michigan, and Dana Farber Cancer Institute (Table 1 and table S1). Each external institution provided demographic, socioeconomic, oncologic, and therapeutic clinical trial screening and enrollment variables, with enrollment defined as enrolling in a therapeutic phase I-IV clinical trial at their respective institutions. The Duke subset consisted of 103 adult glioma patients treated between 20 January 2017 and 4 December 2023. The Michigan subset consisted of 90 adult glioma patients treated between 1 January 2017 and 31 December 2023. The Dana Farber Cancer Institute subset consisted of 37 patients treated between 30 January 2004 and 20 December 2017. For additional context regarding the landscape of clinical trials at each external institution, we sought to determine the number of available clinical trials and enrollment rates during the periods specified above. At Duke, there were 57 therapeutic glioma trials with an average annual enrollment rate of 120 patients. At Michigan, there were 20 available therapeutic glioma trials with an average yearly enrollment rate of 90 patients. Last, at the Dana Farber Cancer Institute, there were 95 available clinical trials with an average annual enrollment rate of 50 patients.

### Statistical analysis

Demographic, socioeconomic, and oncologic characteristics were summarized with descriptive statistics. Differences in categorical and continuous variables between cohorts were assessed using chi-square tests and two-tailed *t* tests as well as Wilcoxon rank sum tests, respectively. Model screening with 10-fold internal cross validation was used to identify the supervised machine learning technique with the best predictive performance for trial enrollment among the nine machine learning algorithms readily available in JMP Pro, finding that the BNN models performed best across cohorts (Supplementary Text). We elected to use *k*-fold cross-validation rather than fixed splitting for internal validation to more reliably estimate model performance by averaging performance metrics across folds and avoid relying on a single test set that may not accurately represent the rest of the development cohort. Predictor screening in JMP Pro 17 was used to identify influential predictors on internal cross-validation. Feature selection with backward elimination was used to optimize BNN model efficiency while maintaining high performance. External validation was performed using conserved model features.

For each BNN model, three hyperbolic tangent function (TanH), linear activation function (Linear), and Gaussian function (Gaussian) nodes were specified in the first layer of the hidden layer structure. To reduce the influence of outliers and heavily skewed distributions, continuous variables were transformed to near normality using the Johnson Su or Johnson Sb distribution for each BNN model. Given that it was hypothesized that most predictor variables were contributing to the predictive ability of the model, the squared method was used for the penalty function. For each model, boosting was performed by specifying 10 component models and a learning rate of 0.1. Nineteen tours were performed for each model corresponding to the number of times that the fitting process was restarted such that each iteration used different random starting points for the parameter estimates. A random number seed was used for each BNN model to create reproducible models.

Predictive performance was assessed by calculating AUC, accuracy, MR, and MCC. Using independent resampled inputs, ranked variables of importance by mean effect and TE were generated for each model. To help explain the directionality of predictors’ influence on enrollment, Shapley values were generated using the permutation SHAP method for each model. Differences in mean Shapley values by predictor variable levels were assessed using two-tailed *t* tests. To ensure no strict violations of the assumption of independence for SHAP analysis, we performed correlation matrix analysis for each model and removed highly correlated predictors [correlation coefficient (*r*) > 0.7] (*42*). We further used principal components analysis to assess the number of principal components needed to explain greater than 80% of total variance in the data.

To assess the association between predictors and clinical trial enrollment, univariate followed by multivariate logistic regression was performed. Predictors with a *P* value of <0.2 on univariate analysis were selected for multivariate logistic regression. To adjust for multiple comparisons and control for the false discovery rate (FDR), the Benjamini-Hochberg adjustment method was used, calculating the adjusted alpha level to be α = 0.030 based on an FDR of 0.05; therefore, *P* values of <0.03 were considered statistically significant in this study (*43*, *44*). JMP Pro 17 and Python were used for all analyses.
