# Supplementary material for: Predicting therapeutic clinical trial enrollment for adult patients with low- and high-grade glioma using supervised machine learning
Source: Sci Adv. 2025 Jun 4;11(23):eadt5708. doi: 10.1126/sciadv.adt5708 (PMC12136030; doi:10.1126/sciadv.adt5708)
Supplement: Supplementary file 1 — Supplementary Text Figs. S1 to S4 Tables S1 to S7 Legend for data S1 [file sciadv.adt5708_sm.pdf]

Science Advances
AAAS

Supplementary Materials for

Predicting therapeutic clinical trial enrollment for adult patients with low- and high-grade glioma using supervised machine learning

Mulki Mehari *et al.*

Corresponding author: Shawn L. Hervey-Jumper, Shawn.Hervey-Jumper@ucsf.edu

Sci. Adv. **11**, eadt5708 (2025)
DOI: 10.1126/sciadv.adt5708

The PDF file includes:

Supplementary Text
Figs. S1 to S4
Tables S1 to S7
Legend for data S1

Other Supplementary Material for this manuscript includes the following:

Data S1

Supplementary Text

Demographic, socioeconomic, and oncologic data collection

Sex, race, employment status, insurance status, occupation, preferred language, interpreter use, in-state vs. out-of-state location, marital status, and patient zip code were extracted from the electronic health record (EHR). Patients' occupations were categorized into major groups according to the International Standard Classification of Occupations (ISCO) after extraction from the EHR. Using the U.S. Census Bureau's 2021 American Community Survey, patients' zip codes were used to determine median household income and the percentage of the population living below the poverty level by zip code inputted at the population level. Distance from the hospital was determined based on the patient's home addresses documented in the EHR. Age at diagnosis and oncologic variables, such as WHO tumor grade, tumor type, tumor hemisphere and lobe, Karnofsky Performance Scale (KPS), and receipt of chemotherapy and radiation, were extracted from the UCSF Brain Tumor Cancer Registry.

UCSF Cohort - Quantification of the extent of resection (EOR)

Pre- and post-operative tumor volumes were quantified using BrainLab Smartbrush software (Brainlab, Munich, Germany). Pre-operative MRI scans were obtained within 24 hours prior to resection, and post-operative scans were all obtained within 72 hours post-resection. Total contrast-enhancing (CE) and non-enhancing (NE) tumor volumes were measured at both pre-operative and post-operative time points. The total CE tumor volume was measured on T1-weighted post-contrast images, and the NE tumor volume was measured on T2 or FLAIR sequences. Manual segmentation was performed with region-of-interest analysis painting" inclusion regions based on fluid-attenuated inversion-recovery (FLAIR) sequences from pre- and post-operative MRI scans to quantify tumor volume. Extent of Resection (EOR) was calculated as: (pre-operative tumor volume - post-operative tumor volume)/(pre-operative tumor volume) x 100%. Multifocal or multicentric disease was defined as noncontiguous areas of disease based on T1-weighted post contrast images or FLAIR sequences. All multifocal lesions were measured separately and summed together for a single volumetric measurement. Volumetric measurements were made blinded to clinical outcomes. To ensure that post-operative FLAIR signal was not surgically induced edema or ischemia, FLAIR pre- and post-operative MRIs were carefully compared alongside DWI sequences prior to including each region in the volume segmentation.

Minority cohort for BNN model development

The minority patients from the development and validation cohorts were aggregated into a pooled minority-only cohort. A BNN model was developed on the pooled cohort, with a holdback proportion of 0.01. As described above, the model parameters and variables of importance calculations for the BNN model were determined.

Correlation matrix analysis and principal component analysis:

Continuous variables were scaled, and categorical variables were one-hot and encoded to binary predictor levels. The correlation coefficients for each predictor pair were generated. Correlation coefficients between predictor pairs greater than 0.7 were considered highly correlated and coefficients greater than 0.4 and less than 0.7 were considered moderately correlated. Highly correlated predictors were removed from our models to reduce redundancy and generate new models. For each PCA analysis, the percentage of variance explained by each principal component was determined to assess for any dominant principal components and the number of

principal components needed to surpass the common threshold of 80% of total variance to evaluate model redundancy. Principal component loadings were generated to assess how individual predictors contributed to each principal component and to explore which features were clustered.

For the whole cohort, on correlation matrix analysis, no variables were highly correlated ($r \geq 0.7$).*(42)* A few variables were moderately correlated ($0.4 \leq r < 0.7$), such as age at diagnosis and WHO grade 4 tumor (r=0.586), age at diagnosis and Medicare insurance (r=0.545), age at diagnosis and retired employment status (r=0.565), and Medicare insurance and retired employment status (r = 0.545). While these values demonstrate a modest correlation between some variables, the correlations do not violate the assumption of independence for SHAP analysis, as each variable still contributes uniquely to the model and is not interchangeable. We proceeded with principal component analysis (with one-hot encoding to transform categorical predictors into numerical representations), finding that 13 principal components were required to explain more than 80% of the variance. These 13 principal components drew from all 11 included variables with no dominant principal component, suggesting that a substantial number of independent dimensions remain.

Similar to the whole cohort, for the women cohort, there were no highly correlated predictors ($r \geq 0.7$). Similar variables were moderately correlated, such as age and Medicare insurance (r=0.590), age and retirement (r=0.560), age and WHO grade 4 tumor (r=0.580), Hispanic or Latino ethnicity and other race (r=0.450), retirement and Medicare insurance (r=0.523), active employment and professional occupation ISCO (r=0.420), and WHO grade 4 tumor and no chemotherapy treatment (r=-0.420). PCA analysis showed 13 principal components required to explain greater than 80% of the variance. These 13 principal components drew from all 12 included predictor variables with no dominant principal component, suggesting that a substantial number of independent dimensions remain.

In contrast, for the minority cohort model, the median household income by zip code and the percentage of households living below the poverty line by zip code were highly correlated (r=-0.711). Therefore, to reduce redundancy, we removed neighborhood poverty levels as a predictor for the minority BNN model. No additional predictors were highly correlated after removing poverty as a predictor ($r \geq 0.7$). Age at diagnosis and Medicare insurance (r=0.549), age at diagnosis and retirement (r=0.612), and Medicare insurance and retirement (r=0.663) were moderately correlated. We performed PCA analysis, finding that 13 principal components were again required to explain more than 80% of the variance. These 13 principal components drew from all 13 predictor variables with no dominant principal component.

Fig. S1. Receiver operator curves (ROC) and ranked variables of importance for boosted neural network (BNN) models for trial screening

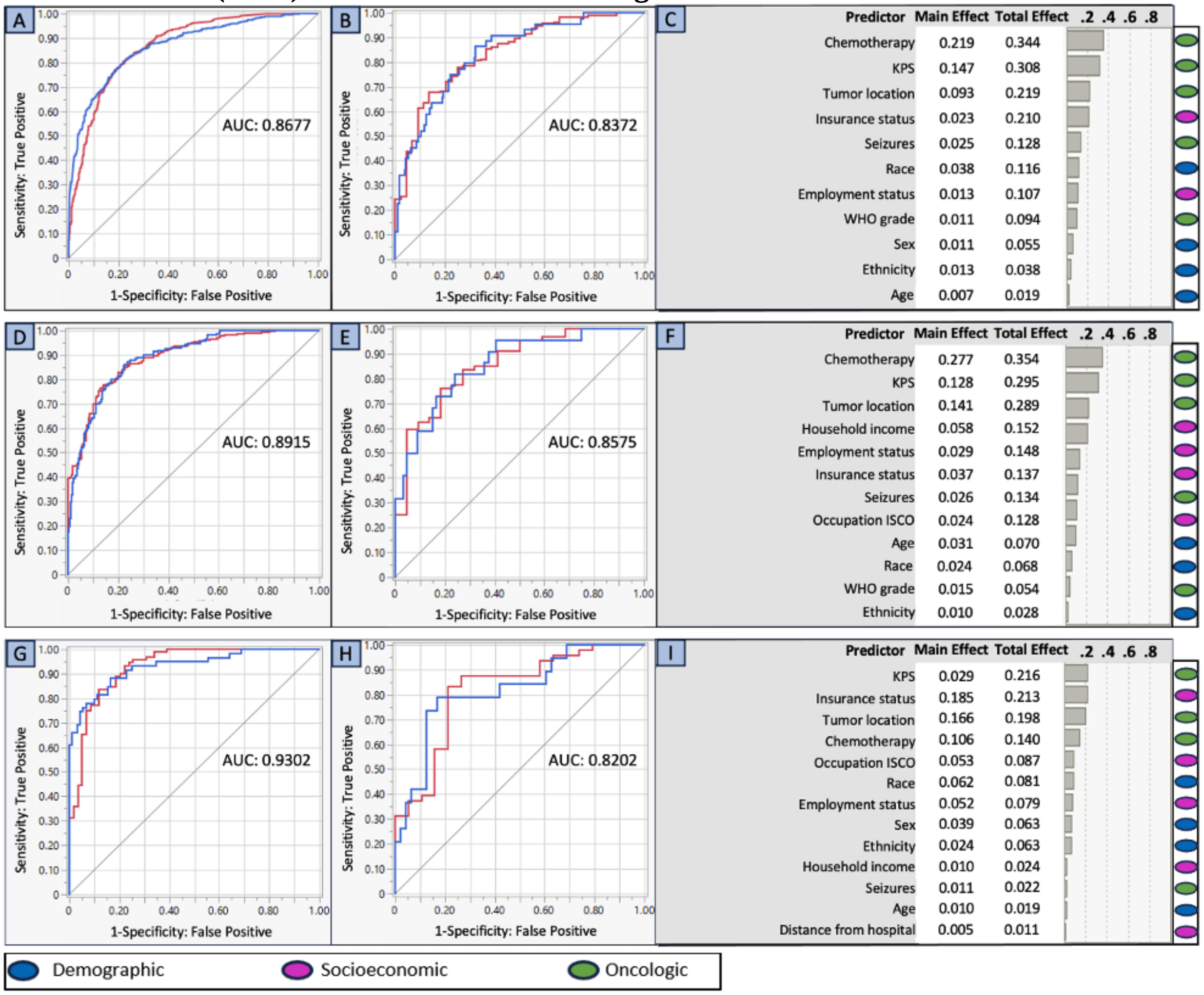

The figure depicts the ROC curves and variables of importance for predicting therapeutic clinical trial screening using BNN models for the whole, women only, and minority only cohorts. In all ROC curves, the red lines represent clinical trial screening while the blue lines represent lack of screening. The area under the curve (AUC) values for each ROC curve are listed. Each variable's main and total effects are listed in variables of importance output for each model. Adjacent to the variables of importance outputs are color-coded heatmaps indicating whether the ranked factors are demographic, socioeconomic, or oncologic variables. A) The ROC curve for the whole development cohort; B) The ROC curve for the whole validation cohort; C) The variables of importance for the whole cohort model in descending order of importance; D) The ROC curve for the women development cohort; E) The ROC curve for the women validation cohort; F) The ranked variables of importance for the women cohort model in descending order of importance; G) The ROC curve for the minority development cohort; H) The ROC curve for the minority validation cohort; I) The ranked variables of importance for the minority cohort model in descending order of importance.

Fig. S2. Receiver operator curve (ROC) and variables of importance for a boosted neural network (BNN) model developed from the minority patients from the development and validation cohorts

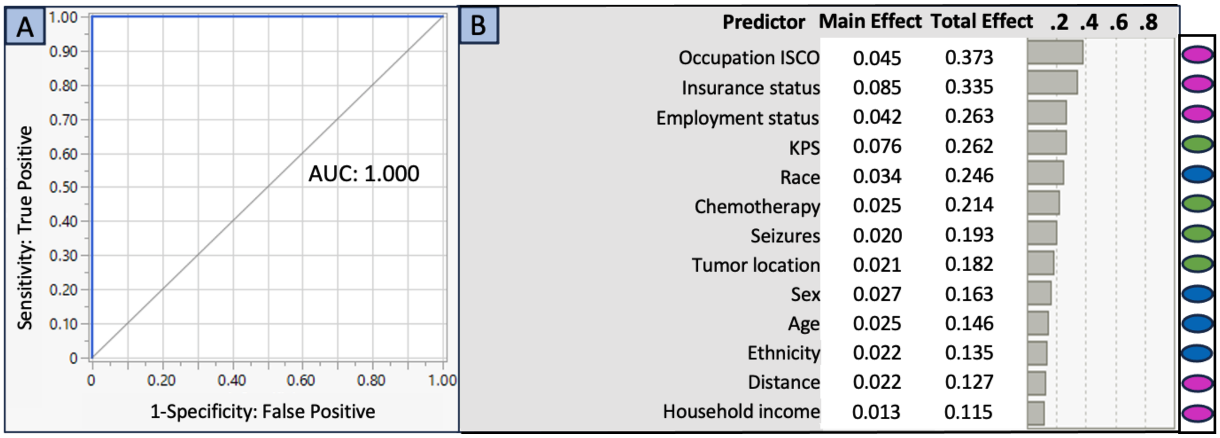

The figure shows the ROC curve and the variables of importance for a boosted neural network (BNN) algorithm that was trained on the combined minority patients from the development and validation cohorts. In the ROC curve, the red line represents clinical trial enrollment while the blue line represents lack of enrollment. The area under the curve (AUC) value for the ROC curve is listed. The main and total effects for each variable are listed in the variables of importance output for the BNN model. Adjacent to the variables of importance outputs are color-coded heatmaps indicating whether the ranked factors are demographic, socioeconomic, or oncologic variables. A) The ROC curve for the BNN algorithm trained on the combined minority cohort B) The variables of importance for the BNN model in descending order of importance.

Fig. S3. Shapley values for influential predictors for the women only cohort boosted neural network (BNN) model

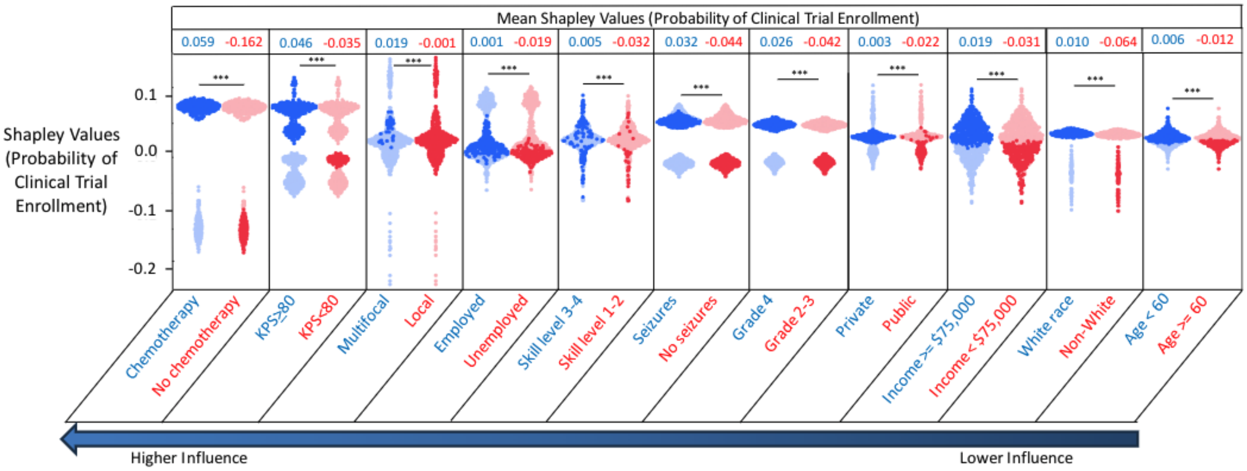

*** p<0.0001

The figure shows the distribution of Shapley values for each influential predictor in the women only cohort BNN model. Each dot corresponds to the Shapley value for each predictor for a single patient. Positive and negative Shapley values indicate increased and decreased probability of enrollment, respectively. Each predictor is stratified into two levels, with the characteristic associated with higher mean Shapley values color-coded in dark blue and the characteristic with the lower mean Shapley values color-coded in dark red. T-tests were used to assess for differences in mean Shapley values for each predictor. Predictors are arranged from most influential to least influential (left to right) according to the variables of importance calculation for the predictive model.

Fig. S4. Shapley values for influential predictors for the minority only cohort boosted neural network (BNN) model

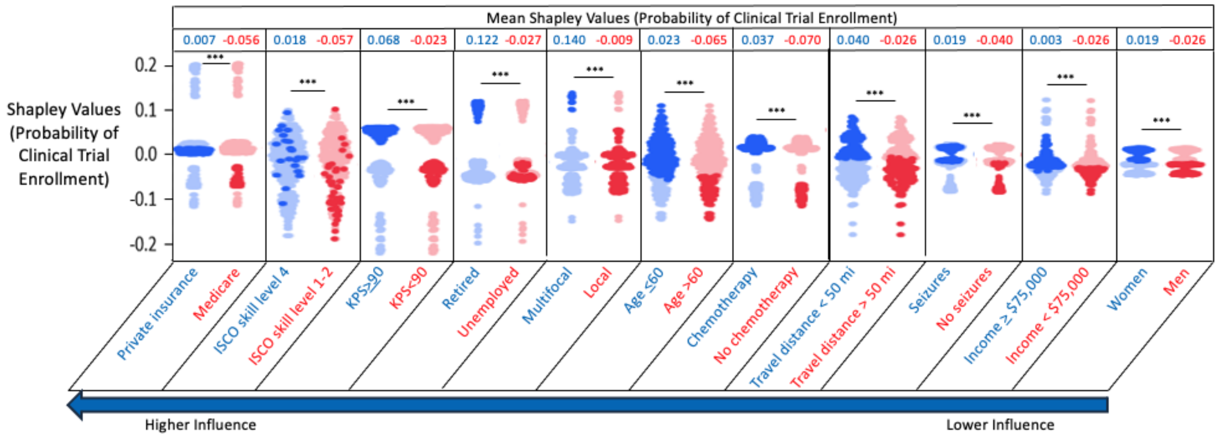

*** p<0.0001

The figure shows the distribution of Shapley values for each influential predictor in the minority only cohort BNN model. Each dot corresponds to the Shapley value for each predictor for a single patient. Positive and negative Shapley values indicate increased and decreased probability of enrollment, respectively. Each predictor is stratified into two levels, with the characteristic associated with higher mean Shapley values color-coded in dark blue and the characteristic with the lower mean Shapley values color-coded in dark red. T-tests were used to assess for differences in mean Shapley values for each predictor. Predictors are arranged from most influential to least influential (left to right) according to the variables of importance calculation for the predictive model.

Table S1: Demographic, socioeconomic, and oncologic characteristics of the Duke University, University of Michigan, and Dana Farber Cancer Institute subsets of the validation cohort

	Combined External Validation Cohort		
	**Duke University Subset**	**University of Michigan Subset**	**Dana Farber Cancer Institute Subset**
	(N = 103)	(N = 90)	(N = 37)
**Mean age (+SD)***	55.7 +15.6	50.4 +17.4	49.2 + 10.5
**Sex**			
Female	40 (38.8%)	31 (34.44%)	18 (48.65%)
Male	63 (61.2%)	59 (65.56%)	19 (51.35%)
**Race**			
Asian	2 (1.9%)	8 (8.89%)	2 (5.41%)
Native Hawaiian and Pacific Islander	0 (0.0%)	0 (0.0%)	0 (0.0%)
Black/African American	13 (12.6%)	10 (11.11%)	4 (10.81%)
American Indians/Alaska Natives	1 (1.0%)	1 (1.11%)	0 (0.0%)
White	81 (78.6%)	63 (70.00%)	26 (70.27%)
Other	3 (2.9%)	7 (7.78%)	4 (10.81%)
Unknown	3 (2.9%)	1 (1.11%)	1 (2.70%)
**Ethnicity**			
Hispanic/Latino	2 (1.9%)	6 (6.67%)	12 (32.43%)
Not Hispanic/Latino	95 (92.2%)	84 (93.33%)	25 (67.57%)
Unknown	6 (5.8%)	0 (0.0%)	0 (0.0%)
**Minority status**			
Minority	19 (18.5%)	28 (31.11%)	19 (51.35%)
Non-minority	78 (75.7%)	62 (68.89%)	18 (48.65%)
Unknown	6 (5.8%)	0 (0.0%)	0 (0.0%)
**Insurance status**			
Private	54 (52.4%)	43 (47.78%)	6 (16.22%)
Medi-cal/Medicaid	4 (3.9%)	18 (20.00%)	0 (0.0%)
Medicare	42 (40.8%)	26 (28.89%)	5 (13.51%)
Unknown	1 (1.0%)	2 (2.2%)	26 (70.3%)
Self-pay	2 (1.9%)	1 (1.1%)	0 (0.0%)
**Employment status**			
Employed	56 (54.4%)	52 (57.78%)	28 (75.68%)
Unemployed	11 (10.7%)	10 (11.11%)	3 (8.11%)
Retired	32 (31.1%)	17 (18.89%)	6 (16.22%)

Unknown	4 (3.9%)	11 (12.22%)	0 (0.0%)
**Median household income (IQR)****	$60657 [52138-78682]	$70331 [58441-91440.5]	$101354 [72286.5-128810.5]
**Median percentage below poverty level (IQR)****	12.2 [8.1-16.5]	8.6 [5.7-13.6]	7.2 [4.4-12.8]
**Median distance from hospital if in-state in miles (IQR)****	159 [42-280]	55.6 [31.45-85.55]	23.5 [8.31-55.55]
**WHO grade**			
WHO grade 2-3	23 (22.3%)	17 (18.89%)	6 (16.22%)
WHO grade 4	80 (77.7%)	73 (81.11%)	31 (83.78%)
**Tumor location: Lobe**			
Brainstem, insular, basal ganglia	8 (7.7%)	0 (0.0%)	2 (5.41%)
Cerebellum	2 (1.9%)	1 (1.11%)	0 (0.0%)
Frontal	41 (39.8%)	29 (32.22%)	18 (48.65%)
Multifocal	2 (1.9%)	0 (0.0%)	0 (0.0%)
Unknown	0 (0.0%)	0 (0.0%)	0 (0.0%)
Occipital	5 (4.9%)	8 (8.89%)	1 (2.70%)
Parietal	12 (11.7%)	11 (12.22%)	8 (21.62%)
Temporal	33 (32.0%)	41 (45.56%)	8 (21.62%)
**Seizure**			
Yes	48 (46.7%)	0 (0.0%)	0 (0.0%)
No	54 (52.4%)	0 (0.0%)	0 (0.0%)
Unknown	1 (1.0%)	90 (100%)	37 (100%)
**Chemotherapy**			
Yes	89 (86.4%)	83 (92.22%)	37 (100%)
No	12 (11.7%)	4 (3.33%)	0 (0.0%)
Unknown	2 (1.9%)	4 (4.44%)	0 (0.0%)
**KPS**			
<80	28 (27.2%)	19 (21.11%)	10 (27.03%)
80	33 (32.0%)	23 (25.56%)	8 (21.62%)
90	37 (35.9%)	38 (42.22%)	14 (37.84%)
100	5 (4.9%)	9 (10.00%)	3 (8.11%)
Unknown	0 (0.0%)	1 (1.11%)	2 (5.41%)
**Screened for a clinical trial**			
Yes	71 (68.9%)	78 (86.67%)	37 (100%)
No	32 (31.1%)	12 (13.33%)	0 (0.0%)
**Enrolled in a clinical trial**			
Yes	30 (29.1%)	68 (75.56%)	37 (100%)

No	73 (70.9%)	22 (24.44%)	0 (0.0%)

Abbreviations: UCSF = University of California, San Francisco, SD = standard deviation, IQR = interquartile range, WHO = World Health Organization, KPS = Karnofsky Performance Scale
*Difference calculated using a two-tailed t-test
**Difference calculated using a Wilcoxon rank-sum test

Table S2. Demographic, socioeconomic, and oncologic characteristics of women and minority subsets of development and validation cohorts

	Women Only Subset of UCSF Development Cohort	Women Only Subset of Combined External Validation Cohort	P-value	Minority Only Subset of UCSF Development Cohort	Minority Only Subset of Combined External Validation Cohort	P-value
	**(n=445)**	**(n=89)**		**(n=151)**	**(n=66)**	
**Mean age (±STD)***	50.6 ± 14.8	54.6 ± 16.7	0.041	47.9 ± 15.5	46.3 ± 16.8	0.501
**Sex**			-			0.357
Female	445 (100.0%)	89 (100.0%)		72 (47.7%)	39 (59.1%)	
Male	0 (0.0%)	0 (0.0%)		79 (52.3%)	27 (40.9%)	
**Race**			<0.0001			<0.0001
Asian	24 (5.0%)	5 (5.6%)		48 (31.8%)	12 (18.2%)	
Native Hawaiian and Pacific Islander	7(1.6%)	0 (0.0%)		17 (11.3%)	0 (0.0%)	
Black/African American	10 (2.3%)	12 (13.5%)		17 (11.3%)	27 (40.9%)	
American Indians/Alaska Natives	2 (0.5%)	1 (1.1%)		4 (2.7%)	2 (3.0%)	
White	387 (87.0%)	65 (73.0%)		36 (23.8%)	10 (15.2%)	
Other	12 (2.7%)	4 (4.5%)		29 (19.2%)	14 (21.2%)	
Unknown	3 (0.7%)	2 (2.3%)		0 (0.0%)	1 (1.5%)	
**Ethnicity**			0.431			0.488
Hispanic/Latino	26 (5.8%)	8 (9.0%)		56 (37.1%)	20 (30.3%)	
Not Hispanic/Latino	413 (92.8%)	79 (88.8%)		94 (62.3%)	46 (69.7%)	
Unknown	6 (1.4%)	2 (2.3%)		1 (0.7%)	0 (0.0%)	
**Minority status**			0.006			-
Minority	72 (16.2%)	27 (30.3%)		151 (100.0%)	66 (100.0%)	
Non-minority	36 (82.3%)	60 (67.4%)		0 (0.0%)	0 (0.0%)	
Unknown	7 (1.6%)	2 (2.3%)		0 (0.0%)	0 (0.0%)	
**Preferred language**						
English	424 (95.3%)			118 (78.2%)		
Not English	17 (3.8%)			32 (21.2%)		
Unknown	4 (0.9%)			1 (0.7%)		
**Interpreter used**						

Yes	11 (2.5%)			25 (16.6%)		
No	430 (96.6%)			121 (80.1%)		
Unknown	4 (0.9%)			5 (3.3%)		
**Insurance status**			0.014			0.174
Private	285 (64.0%)	40 (44.9%)		77 (51.0%)	24 (36.4%)	
Medi-cal/Medicaid	36 (8.1%)	8 (9.0%)		26 (17.2%)	14 (21.2%)	
Medicare	81 (18.2%)	26 (29.2%)		23 (15.2%)	17 (25.8%)	
Unknown	38 (8.5%)	13 (14.6%)		19 (12.6%)	10 (15.2%)	
Self-pay	5 (1.1%)	2 (2.3%)		6 (4.0%)	1 (1.5%)	
**Employment status**			<0.0001			0.103
Employed	171 (38.4%)	49 (55.1%)		66 (43.7%)	38 (57.6%)	
Unemployed	155 (34.8%)	10 (11.2%)		54 (35.8%)	13 (19.7%)	
Retired	104 (23.4%)	23 (25.8%)		27 (17.9%)	12 (18.2%)	
Unknown	15 (3.4%)	7 (7.9%)		4 (2.7%)	3 (4.6%)	
**Median household income (IQR)****	93274 (66617-125386)	65796 (53661-101538)	<0.0001	93794 (66145-131032)	71602 (556128-9437)	<0.0001
**Median percentage below poverty level (IQR)****	8.7 (5.9-13.8)	9.9 (5.8-17.1)	0.050	9.9 (6.3-14.8%)	10 (6.2-16.2)	0.277
**Marital status**						
Married/Registered Domestic Partner/Significant Other	298 (67.0%)			98 (64.9%)		
Divorced/Separated	25 (5.6%)			9 (6.0%)		
Widowed	27 (6.1%)			2 (1.3%)		
Single	93 (20.9%)			42 (27.8%)		
Unknown	2 (0.5%)			0 (0.0%)		
**Location**						
In-state	345 (77.5%)			131 (86.8%)		
Out-of-state	96 (21.6%)			15 (9.9%)		
International	4 (0.9%)			5 (3.1%)		
**Median distance from hospital if in-state in miles (IQR)****	76.4 (31.9-167.0)	59.4 (20.2-165.5)	0.152	56.5 (18.5-111.5)	46.4 (22.7-142.5)	0.113
**Tumor type**						
WHO grade 2-3 astrocytoma	88 (19.8%)			35 (23.2%)		

WHO grade 2-3 oligodendroglioma	85 (19.1%)			39 (25.8%)		
WHO grade 4 IDH mutant astrocytoma	16 (3.6%)			7 (4.6%)		
WHO grade 4 glioblastoma	256 (57.5%)			70 (46.4%)		
**WHO grade**			0.001			0.019
WHO grade 2-3	173 (38.9%)	18 (20.2%)		74 (49.0%)	21 (31.8%)	
WHO grade 4	272 (61.1%)	71 (79.8%)		77 (51.0%)	45 (68.2%)	
**Tumor location: hemisphere**						
Left	223 (50.1%)			76 (50.3%)		
Right	208 (46.7%)			72 (47.7%)		
Bilateral	2 (0.5%)			1 (0.7%)		
NA	12 (2.7%)			2 (1.3%)		
**Tumor location: lobe**			0.001			0.086
Brainstem, insular, basal ganglia	36 (8.1%)	0 (0.0%)		16 (10.6%)	3 (4.6%)	
Cerebellum	1 (0.2%)	2 (2.3%)		0 (0.0%)	1 (1.5%)	
Frontal	193 (43.4%)	37 (41.6%)		68 (45.0%)	32 (48.5%)	
Multifocal	34 (7.6%)	2 (2.3%)		9 (6.0%)	0 (0.0%)	
Unknown	13 (2.9%)	0 (0.0%)		2 (1.3%)	0 (0.0%)	
Occipital	9 (2.0%)	2 (2.3%)		4 (2.7%)	0 (0.0%)	
Parietal	60 (13.5%)	17 (19.1%)		21 (13.9%)	11 (16.7%)	
Temporal	99 (22.3%)	29 (32.6%)		31 (20.5%)	19 (28.8%)	
**Seizure**			<0.0001			<0.0001
Yes	245 (55.1%)	16 (18.0%)		97 (64.2%)	9 (13.6%)	
No	190 (42.7%)	24 (27.0%)		49 (32.5%)	10 (15.2%)	
Unknown	10 (2.3%)	49 (55.1%)		5 (3.3%)	47 (71.2%)	
**Chemotherapy**			0.002			0.001
Yes	324 (72.8%)	78 (87.6%)		96 (63.6%)	57 (86.4%)	
No	110 (24.7%)	7 (7.9%)		47 (31.1%)	5 (7.6%)	
Unknown	11 (2.5%)	4 (4.5%)		8 (5.3%)	4 (6.1%)	
**Radiation**						
Yes	306 (68.8%)			37 (24.5%)		
No	116 (26.1%)			9 (6.0%)		
Unknown	23 (5.2%)			105 (69.5%)		
**KPS**			<0.0001			0.048
<80	66 (14.8%)	25 (28.1%)		28 (18.5%)	17 (25.8%)	

80	76 (17.1%)	23 (25.8%)		24 (15.9%)	15 (22.7%)	
90	166 (37.3%)	32 (36.0%)		68 (45.0%)	28 (42.4%)	
100	12 (2.7%)	6 (6.7%)		6 (4.0%)	4 (6.1%)	
Unknown	125 (28.1%)	3 (3.4%)		25 (16.6%)	2 (3.0%)	
**Screened for a clinical trial**			0.010			0.146
Yes	271 (60.9%)	67 (75.3%)		92 (60.9%)	47 (71.2%)	
No	174 (39.1%)	22 (24.7%)		59 (39.1%)	19 (28.8%)	
**Enrolled in a clinical trial**			<0.0001			0.002
Yes	144 (32.4%)	52 (58.4%)		41 (27.2%)	31 (48.5%)	
No	301 (67.6%)	37 (41.6%)		110 (72.9%)	34 (51.5%)	
**Number of clinical trials discussed during screening**						
0 trials discussed	168 (37.8%)			58 (38.4%)		
1-2 trials discussed	230 (51.7%)			78 (51.7%)		
3-4 trials discussed	23 (5.2%)			6 (4.0%)		
5+ trials discussed	6 (1.4%)			0 (0.0%)		
Unspecified number of trials discussed	18 (4.0%)			9 (6.0%)		

Abbreviations: UCSF = University of California, San Francisco, SD = standard deviation, IQR = interquartile range, WHO = World Health Organization, KPS = Karnofsky Performance Scale, IDH= isocitrate dehydrogenase

*Difference calculated using a two-tailed t-test

**Difference calculated using a Wilcoxon rank-sum test

Table S3. Univariate and multivariate logistic regression for enrollment among those screened for a clinical trial

	Univariate Logistic Regression		Multivariate Logistic Regression	
	OR (95% CI)	P-value	OR (95% CI)	P-value
**Age**	1.035 (0.929-1.153)	0.537		
**Sex**		0.254		
Female	0.831 (0.605-1.142)	0.254		
Male	Referent			
**Race**		0.086		
White	Referent			
Asian	0.582 (0.295-1.148)	0.119		
Native Hawaiian and Pacific Islander	0.728(0.208-2.543)	0.619		
Black/African American	0.323 (0.098-1.063)	0.063		
American Indian/Alaska Native	1072208 (0-*)	0.991		
Other race(s)	0.485 (0.170-1.382)	0.486		
Unknown	0.291(0.056-1.513)	0.142		
**Ethnicity**		0.488		
Hispanic/Latino	0.777 (0.364-1.659)	0.515		
Not Hispanic/Latino	Referent			
Unknown	0.518 (0.145-1.855)	0.312		
**Minority status**		0.024		
Minority	0.58 (0.371-0.906)	0.017		
Non-minority	Referent			
Unknown	0.412 (0.119-1.425)	0.162		
**Preferred language**		0.293		
English	Referent			
Not English	0.434 (0.144-1.310)	0.139		
Unknown	1.563 (0.141-17.325)	0.716		
**Interpreter used**		0.287		
Yes	Referent			
No	1.257 (0.401-3.942)	0.695		
Unknown	1473120 (0.000-*)	0.987		

**Insurance status**		0.0002		0.002
Private	Referent		Referent	
Medi-cal/Medicaid	0.534 (0.287-0.996)	0.049	0.651 (0.296-1.432)	0.286
Medicare	0.533 (0.344-0.826)	0.005	0.475 (0.248-0.909)	0.025
Unknown	2.499 (1.276-4.893)	0.008	3.811 (1.517-9.573)	0.004
Self-pay	0.975 (0.216-4.412)	0.974	14644114748 (0.000-*)	1.000
**Employment status**		0.195		
Employed	Referent			
Unemployed	0.961 (0.653-1.415)	0.840		
Retired	1.011 (0.958-0.678)	1.508		
Unknown	0.353 (0.131-0.953)	0.040		
**Occupational status - ISCO code**		0.406		
1 - Managers	Referent			
2 - Professionals	1.696 (0.746-3.856)	0.207		
3 - Technicians and associate professionals	1.364 (0.494-3.766)	0.550		
4 - Clerical support workers	4.167 (0.438-39.68)	0.215		
5 - Service and sales workers	1.019 (0.334-3.109)	0.974		
6 - Skilled agricultural, forestry, and fishery workers	0.833 (0.146-4.752)	0.837		
7 - Craft and related trades worker	0.417 (0.067-2.599)	0.349		
8 - Plant and machine operators, and assemblers	0.000 (0.000-*)	0.991		
9 - Elementary occupations	1227600 (0.016-*)	0.991		
0 - Armed forces occupations	0.833 (0.048-14.482)	0.900		
Other: self-employed	0.938 (0.290-3.030)	0.914		
On disability	0.972 (0.347-2.727)	0.957		
Student	1227600 (0.000-*)	0.991		
Unknown	0.946 (0.464-1.930)	0.879		
**Household income**	1.051 (1.010-1.094)	0.006		

**Percentage below poverty level**	0.980 (0.954-1.006)	0.135		
**Marital status**		0.334		
Married/Registered Domestic Partner/Significant Other	Referent			
Divorced/Separated	1.477 (0.696-3.134)	0.310		
Widowed	1.392 (0.459-4.220)	0.559		
Single	0.749 (0.503-1.115)	0.154		
Unknown	2.320 (0.240-22.476)	0.468		
**Location**		0.019*		
In-state	Referent			
Out-of-state	0.736 (0.502-1.079)	0.116		
International	0.181 (0.038-0.863)	0.032		
**Distance from UCSF if in state in miles**	0.976 (0.961-0.991)	0.002		
**Tumor type**		0.046		
WHO grade 2-3 astrocytoma	Referent			
WHO grade 2-3 oligodendroglioma	1.339 (0.795-2.256)	0.273		
WHO grade 4 IDH mutant astrocytoma	1.69 (0.745-3.832)	0.209		
WHO grade 4 glioblastoma	1.769 (1.176-2.663)	0.006		
**WHO grade**		0.009		0.019
WHO grade 2-3	Referent		Referent	
WHO grade 4	1.544 (1.113-2.142)	0.009	2.024 (1.127-3.636)	0.019
**Tumor location: hemisphere**		0.644		
Left	Referent			
Right	0.992 (0.722-1.363)	0.958		
Bilateral	1.184 (0.195-7.178)	0.854		
Unknown	0.000 (0.000-*)	0.991		
**Tumor location: lobe**		0.540		
Brainstem, insular, basal ganglia, or thalamus	1.324 (0.739-2.372)	0.345		
Frontal	Referent			
Multifocal	1.257 (0.724-2.181)	0.416		

Occipital	1.958 (0.587-6.529)	0.274		
Parietal	1.397 (0.867-2.250)	0.169		
Temporal	0.907 (0.601-1.368)	0.642		
Unknown	0.87 (0.054-14.072)	0.922		
**Seizure**		0.153		0.042
Yes	1.379 (0.988-1.925)	0.059	1.744 (1.121-2.713)	0.014
No	Referent		Referent	
Unknown	0.972 (0.330-2.867)	0.96	1.994 (0.527-7.549)	0.310
**Preoperative tumor volume in mL (%)**		0.261		
<25	Referent			
25-49	1.494 (0.942-2.370)	0.088		
50-99	1.639 (1.038-2.589)	0.034		
100-400	1.244 (0.789-1.963)	0.347		
Unknown	1.061 (0.065-17.305)	0.967		
**Volumetric extent of resection (%)**		0.534		
<80	1.173 (0.847-1.627)	0.288		
>=80	Referent			
Unknown	0.29 (0.03-2.832)	0.287		
**Chemotherapy**		0.002		
Yes	Referent			
No	0.411 (0.265-0.637)	<.0001		
Unknown	0.567 (0.171-1.883)	0.354		
**Radiation**		0.079		
Yes	Referent			
No	0.639 (0.432-0.945)	0.025		
Unknown	0.996 (0.393-2.519)	0.992		
**KPS**		0.008		
<80	Referent			
>=80	2.106 (1.284-3.454)	0.003		
Unknown	1.565 (0.863-2.839)	0.140		
**Number of clinical trials discussed**		<0.0001		<0.0001
1 trial discussed	Referent		Referent	
2 trials discussed	1.960 (1.344-2.858)	0.001	2.101 (1.336-3.302)	0.001
3 trials discussed	2.250 (1.237-4.093)	0.008	2.201 (1.107-4.377)	0.025

4 trials discussed	2.170 (0.862-5.465)	0.100	1.148 (0.418-3.148)	0.789
5+ trials discussed	8.608 (1.956-37.872)	0.004	5.073 (1.065-24.174)	0.042
Unspecified number of trials discussed	0.000 (0.000-*)	0.986	0.000 (0.000-*)	1.000

Abbreviations: OR = odds ratio, CI = confidence interval, ISCO = International Standard Classification of Occupations, UCSF = University of California, San Francisco, WHO = World Health Organization, KPS = Karnofsky Performance Scale, IDH= isocitrate dehydrogenase

Table S4. Association between sex, minority status, and preferred language and the number of clinical trials discussed with patients during trial screening

	Number of Clinical Trials Discussed with Patients During Trial Screening									Mean+/-Standard Deviation	P-value
	1	2	3	4	5	6	7	8	Total		
**Sex***											0.001
Female	151 (59.2%)	75 (29.4%)	16 (6.3%)	7 (2.8%)	4 (1.6%)	1 (0.4%)	1 (0.4%)	0 (0.0%)	255 (43.2%)	1.61 +/- 0.96	
Male	158 (47.2%)	107 (31.9%)	42 (12.5%)	15 (4.5%)	5 (1.5%)	7 (2.1%)	0 (0.0%)	1 (0.3%)	335 (56.8%)	1.89 +/- 1.16	
**Minority status****											0.0002
Minority	49 (58.3%)	29 (34.5%)	6 (7.1%)	0 (0.0%)	0 (0.0%)	0 (0.0%)	0 (0.0%)	0 (0.0%)	84 (14.5%)	1.49 +/- 0.63	
Non-minority	257 (51.7%)	148 (29.8%)	51 (10.3%)	22 (4.4%)	9 (1.8%)	8 (1.6%)	1 (0.2%)	1 (0.2%)	497 (85.5%)	1.81 +/- 1.14	
**Preferred language*****											0.002
English	299 (52.0%)	178 (98.3%)	57 (9.9%)	22 (3.8%)	9 (1.6%)	8 (1.4%)	1 (0.2%)	1 (0.2%)	575 (98.0%)	1.78 +/- 1.09	
Not English	9 (75.0%)	3 (25.0%)	0 (0.0%)	0 (0.0%)	0 (0.0%)	0 (0.0%)	0 (0.0%)	0 (0.0%)	12 (2.0%)	1.25 +/- 0.45	

Among all patients with known *sex, **minority status, and ***preferred language and a specified number of clinical trials discussed during screening

Table S5. Univariate and multivariate logistic regression for screening for a clinical trial among all patients in the development cohort

	Univariate Logistic Regression		Multivariate Logistic Regression	
	**OR (95% CI)**	**P-value**	**OR (95% CI)**	**P-value**
**Age**	0.931 (0.856-1.012)	0.092		
**Sex**		0.720		
Female	0.955 (0.743-1.228)	0.720		
Male	Referent			
**Race**		0.110		
White	Referent			
Asian	2.011 (1.033-3.917)	0.040		
Native Hawaiian and Pacific Islander	0.838 (0.328-2.143)	0.712		
Black/African American	2.179 (0.071-6.734)	0.176		
American Indian/Alaska Native	0.223 (0.023-2.156)	0.195		
Other race(s)	0.718 (0.3431.506)	0.381		
Unknown	0.670 (0.233-1.927)	0.458		
**Ethnicity**		0.100		
Hispanic/Latino	0.612 (0.358-1.045)	0.072		
Not Hispanic/Latino	Referent			
Unknown	0.576 (0.242-1.37)	0.212		
**Minority status**		0.473		
Minority	1.019 (0.715-1.452)	0.918		
Non-minority	Referent			
Unknown	0.599 (0.261-1.373)	0.226		

**Preferred language**		0.0028
English	Referent	
Not English	0.338 (0.175-0.656)	0.0013
Unknown	0.471 (0.105-2.118)	0.327
**Interpreter used**		0.153
Yes	Referent	
No	2.374 (1.131-4.982)	0.0223
Unknown	0.375 (0.068-2.08)	0.262
**Insurance status**		0.0002
Private	Referent	
Medi-cal/Medicaid	0.629 (0.396-0.997)	0.0487
Medicare	0.692 (0.495-0.968)	0.0315
Unknown	0.42 (0.283-0.624)	<0.0001
Self-pay	0.531 (0.184-1.533)	0.2418
**Employment status**		0.0442
Employed	Referent	
Unemployed	0.75 (0.554-1.016)	0.063
Retired	0.799 (0.583-1.095)	0.163
Unknown	0.463 (0.245-0.874)	0.0175
**Occupational status - ISCO code**		0.398
1 - Managers	Referent	
2 - Professionals	1.096 (0.567-2.12)	0.785
3 - Technicians and associate professionals	1.098 (0.481-2.507)	0.824
4 - Clerical support workers	0.455 (0.138-1.502)	0.196
5 - Service and sales workers	0.758 (0.32-1.791)	0.527

6 - Skilled agricultural, forestry, and fishery workers	1.212 (0.272-5.395)	0.801		
7 - Craft and related trades worker	0.727 (0.196-2.696)	0.634		
8 - Plant and machine operators, and assemblers	0.606 (0.036-10.238)	0.728		
9 - Elementary occupations	0.152 (0.016-1.453)	0.102		
0 - Armed forces occupations	0.303 (0.051-1.808)	0.19		
Other: self-employed	1.145 (0.429-3.052)	0.787		
On disability	1.97 (0.748-5.184)	0.17		
Student	0.606 (0.036-10.238)	0.728		
Unknown	0.88 (0.495-1.566)	0.664		
**Household income**	1.062 (1.03-1.094)	<0.0001	1.070 (1.017-1.125)	0.008
**Percentage below poverty level**	0.971 (0.951-0.991)	0.0045		
**Marital status**		0.118		
Married/Registered Domestic Partner/Significant Other	Referent			
Divorced/Separated	0.683 (0.404-1.153)	0.154		
Widowed	0.433 (0.215-0.872)	0.0190		
Single	0.936 (0.682-1.284)	0.682		
Unknown	1.238 (0.225-6.803)	0.806		
**Location**		0.407		
In-state	Referent			
Out-of-state	0.83 (0.619-1.114)	0.215		
International	1.267 (0.429-3.742)	0.668		
**Distance from UCSF if in state in miles**	0.988 (0.977-1.000)	0.057		

**Tumor type**		0.361		
WHO grade 2-3 astrocytoma	Referent			
WHO grade 2-3 oligodendroglioma	0.760 (0.505-1.144)	0.188		
WHO grade 4 IDH mutant astrocytoma	1.321 (0.646-2.698)	0.446		
WHO grade 4 glioblastoma	0.890 (0.64-1.238)	0.490		
**WHO grade**		0.750		
WHO grade 2-3	Referent			
WHO grade 4	1.043 (0.806-1.348)	0.75		
**Tumor location: hemisphere**		<0.0001		
Left	Referent			
Right	0.821 (0.636-1.059)	0.129		
Bilateral	2249508 (0.000-*)	0.987		
Unknown	0.019 (0.003-0.138)	<0.0001		
**Tumor location: lobe**		<0.0001		
Brainstem, insular, basal ganglia or thalamus	1.355 (0.832-2.206)	0.222		
Frontal	Referent			
Multifocal	1.72 (1.049-2.821)	0.0317		
Occipital	0.801 (0.350-1.830)	0.598		
Parietal	0.919 (0.641-1.318)	0.646		
Temporal	1.26 (0.898-1.767)	0.182		
Cerebellum	0.000 (0.000-*)	0.985		
Unknown	0.045 (0.011-0.192)	<0.0001		
**Seizure**		0.0004		0.011
Yes	1.672 (1.294-2.161)	<0.0001	1.455 (1.058-2.002)	0.021
No	Referent		Referent	

Unknown	1.131 (0.501-2.55)	0.767	0.907 (0.336-2.453)	0.481
**Preoperative tumor volume in mL (%)**		0.209		
<25	Referent			
25-49	1.169 (0.802-1.704)	0.416		
50-99	0.850 (0.596-1.213)	0.370		
100-400	0.881 (0.615-1.262)	0.490		
Unknown	931532 (0.000-*)	0.987		
**Volumetric extent of resection (%)**		0.534		
<80	0.924 (0.713-1.197)	0.549		
>=80	Referent			
Unknown	2.517 (0.279-22.742)	0.411		
**Chemotherapy**		<0.0001		<0.0001
Yes	Referent		Referent	
No	0.284 (0.212-0.381)	<0.0001	0.239 (0.155-0.370)	<0.0001
Unknown	0.225 (0.107-0.471)	<0.0001	0.303 (0.120-0.765)	0.011
**Radiation**		<0.0001		
Yes	Referent			
No	0.561 (0.419-0.75)	<0.0001		
Unknown	0.265 (0.15-0.469)	<0.0001		
**KPS**		<0.0001		<0.0001
<80	Referent		Referent	
>=80	2.861 (1.988-4.117)	<0.0001	2.286 (1.485-3.517)	<0.0001
Unknown	0.618 (0.415-0.92)	0.018	0.876 (0.529-1.449)	0.606

Abbreviations: OR = odds ratio, CI = confidence interval, NIH = National Institute of Health, ISCO = International Standard Classification of Occupations, UCSF = University of California, San Francisco, WHO = World Health Organization, KPS = Karnofsky Performance Scale, IDH= isocitrate dehydrogenase

Table S6. Univariate and multivariate logistic regression for clinical trial enrollment for the women only and minority only subsets of the development cohort

	Women Only Cohort				Minority Only Cohort			
	Univariate Logistic Regression		Multivariate Logistic Regression		Univariate Logistic Regression		Multivariate Logistic Regression	
	OR (95% CI)	P-value	OR (95% CI)	P-value	OR (95% CI)	P-value	OR (95% CI)	P-value
**Age**	0.989 (0.970-1.008)	0.267			0.779 (0.608-0.999)	0.043		
**Race**		0.108				0.877		
White	Referent				Referent			
Asian	0.988 (0.412 - 2.370)	0.979			1.500 (0.572-3.933)	0.410		
Native Hawaiian and Pacific Islander	0.000 (0.000-*)	0.990			1.250 (0.345-4.529)	0.734		
Black/African American	0.494 (0.103-2.361)	0.377			0.923 (0.239-3.564)	0.908		
American Indian/Alaska Native	0.000 (0.000-*)	0.992			1.000 (0.092-10.865)	1		
Other race(s)	0.988 (0.292-3.344)	0.985			0.783 (0.242-2.53)	0.682		
Unknown	0.000 (0.000-*)	0.990			N/A	N/A		
**Ethnicity**		0.551				0.643		
Hispanic/Latino	1.316 (0.581-2.977)	0.510			0.827 (0.390-1.755)	0.621		
Not Hispanic/Latino	Referent				Referent			
Unknown	0.421 (0.049-3.640)	0.432			0.000 (0.000-*)	0.991		
**NIH-designated minority status**		0.337						

Minority	0.760 (0.434-1.330)	0.336		
Non-minority	Referent			
Unknown	0.430 (0.122-1.521)	0.306		
**Sex**				0.869
Male			Referent	
Female			1.062 (0.518-2.177)	0.869
**Preferred language**		0.076		0.057
English	Referent		Referent	
Not English	0.430 (0.122-1.521)	0.191	0.313 (0.102-0.956)	0.0415
Unknown	0.000 (0.000-*)	0.989	0.000 (0.000-*)	0.991
**Interpreter used**		0.192		0.119
Yes	Referent		Referent	
No	1.301 (0.340-4.979)	0.701	1.694 (0.59-4.864)	0.327
Unknown	0.000 (0.000-*)	0.989	0.000 (0.000-*)	0.992
**Insurance status**		0.688		0.11
Private	Referent		Referent	
Medi-cal/Medicaid	0.723 (0.335-1.559)	0.408	0.865 (0.32-2.338)	0.775
Medicare	0.745 (0.434-1.280)	0.286	0.224 (0.048-1.033)	0.055
Unknown	0.765 (0.364-1.608)	0.480	1.708 (0.608-4.798)	0.310
Self-pay	0.470 (0.052-4.260)	0.502	0.470 (0.052-4.245)	0.501
**Employment status**		0.093		0.437
Employed	Referent		Referent	

Unemployed	0.793 (0.494-1.271)	0.335		1.123 (0.506-2.49)	0.776	
Retired	1.302 (0.785-2.158)	0.307		0.933 (0.338-2.581)	0.894	
Unknown	0.308 (0.067-1.410)	0.129		0.000 (0.000-*)	0.993	
**Occupational status - ISCO code**		0.836			0.579	
1 - Managers	Referent			Referent		
2 - Professionals	0.629 (0.212-1.862)	0.402		4.375 (0.407-47.017)	0.223	
3 - Technicians and associate professionals	1.000 (0.276-3.625)	1.000		3.75 (0.274-51.373)	0.322	
4 - Clerical support workers	0.825 (0.151-4.500)	0.824		0.000 (0.000-*)	0.995	
5 - Service and sales workers	0.491 (0.125-1.929)	0.308		1.000 (0.048-20.829)	1.000	
6 - Skilled agricultural, forestry, and fishery workers	0.000 (0.000-*)	0.992		0.000 (0.000-*)	0.995	
7 - Craft and related trades worker				5.000 (0.150-166.589)	1.000	
8 - Plant and machine operators, and assemblers				0.000 (0.000-*)	0.995	
9 - Elementary occupations	0.000 (0.000-*)	0.994		0.000 (0.000-*)	0.368	
0 - Armed forces occupations				0.000 (0.000-*)	0.997	
Other: self-employed	1.375 (0.158-11.937)	0.773		1.667 (0.074-37.728)	0.748	
On disability	0.688 (0.152-3.102)	0.626		1.429 (0.1-20.437)	0.793	

Student	0.000 (0.000-*)	0.994				
Unknown	0.629 (0.249-1.638)	0.351		1.838 (0.205-16.514)	0.587	
**Household income**	1.040 (1.000-1.090)	0.054		1.068 (0.988-1.154)	0.099	
**Percentage below poverty level**	0.985 (0.953-1.018)	0.352		0.958 (0.899-1.021)	0.172	
**Marital status**		0.784			0.063	
Married, Registered Domestic Partner, Significant Other	Referent			Referent		
Divorced, Separated	0.616 (0.239-1.591)	0.317		0.000 (0.000-*)	0.993	
Widowed	0.821 (0.347-1.941)	0.654		0.000 (0.000-*)	0.997	
Single	0.84 (0.508-1.391)	0.498		0.952 (0.429-2.113)	0.903	
Unknown	1.95 (0.121-31.508)	0.638				
**Location**		0.007			0.668	
In-state	Referent			Referent		
Out-of-state	0.505 (0.297-0.86)	0.019		0.660 (0.176-2.475)	0.538	
International	0.000 (0.000-*)	0.988		1.759 (0.282-10.965)	0.545	
**Distance from UCSF if in state in miles**	.981 (0.962-1.001)	0.051		0.978 (0.943-1.015)	0.209	
**Tumor type**		0.375			0.813	
WHO grade 2-3 astrocytoma	Referent			Referent		

WHO grade 2-3 oligodendroglioma	1.245 (0.642-2.416)	0.516		0.862 (0.309-2.407)	0.777	
WHO grade 4 IDH mutant astrocytoma	2.198 (0.734-6.579)	0.159		1.875 (0.354-9.93)	0.460	
WHO grade 4 glioblastoma	1.480 (0.862-2.543)	0.155		0.865 (0.349-2.146)	0.755	
**WHO grade**		0.145			0.973	
WHO grade 2-3	Referent			Referent		
WHO grade 4	1.358 (0.898-2.055)	0.147		1.013 (0.494-2.075)	0.973	
**Tumor location: hemisphere**		0.003			0.272	
Left	Referent			Referent		
Right	0.899 (0.601-1.344)	0.604		0.939 (0.454-1.941)	0.865	
Bilateral	21053847 (0.000-*)	0.994		10487610 (0.000-*)	0.994	
Unknown	0.000 (0.000-*)	0.987		0.000 (0.000-*)	0.992	
**Tumor location: lobe**		0.234			0.619	
Brainstem, insular, basal ganglia	1.690 (0.820-3.485)	0.155		1.547 (0.494-4.850)	0.454	
Frontal	Referent			Referent		
Multifocal	1.479 (0.701-3.121)	0.304		2.063 (0.500-8.514)	0.317	
Occipital	1.056 (0.256-4.364)	0.94		0.860 (0.084-8.785)	0.899	
Parietal	0.979 (0.526-1.824)	0.947		0.607 (0.181-2.037)	0.419	
Temporal	0.875 (0.516-1.484)	0.621		0.752 (0.278-2.034)	0.575	
Cerebellum	0.000 (0.000-*)	0.991				

Unknown	0.176 (0.022-1.385)	0.099			0.000 (0.000-*)	0.995
**Seizure**		0.032		0.0194		0.065
Yes	1.730 (1.146-2.634)	0.009	2.000 (1.223-3.269)	0.0057	1.832 (0.811-4.14)	0.146
No	Referent		Referent		Referent	
Unknown	1.233 (0.258-4.626)	0.771	0.414 (0.399-9.325)	0.414	0.000 (0.000-*)	0.992
**Preoperative tumor volume in mL**		0.194				0.609
<25	Referent				Referent	
25-49	1.482 (0.817-2.688)	0.196			1.887 (0.63-5.648)	0.256
50-99	1.692 (0.944-3.033)	0.077			1.904 (0.614-5.9)	0.265
100-400	1.184 (0.652-2.151)	0.579			1.904 (0.614-5.9)	0.265
Unknown	4136069 (0.000-*)	0.990			*	*
**Volumetric extent of resection (%)**		0.454				0.681
<80	1.279 (0.837-1.954)	0.255			1.166 (0.56-2.425)	0.682
≥80	Referent				Referent	
Unknown	2.467 (0.151-40.295)	0.526			*	*
**Chemotherapy**		<0.0001		<0.0001		0.144
Yes	Referent		Referent		Referent	
No	0.188 (0.099-0.355)	<0.0001	0.145 (0.062-0.336)	<0.0001	0.451 (0.188-1.082)	0.075
Unknown	0.875 (0.251-3.049)	0.834	1.530 (0.382-6.126)	0.834	1.320 (0.296-5.886)	0.716
**Radiation**		0.007				0.604

Yes	Referent				Referent		
No	0.493 (0.301-0.809)	0.005			0.659 (0.271-1.6)	0.357	
Unknown	0.474 (0.171-1.313)	0.151			0.682 (0.134-3.469)	0.645	
**KPS**		<0.0001		0.006		0.034	
<80	Referent		Referent		Referent		
≥80	2.357 (1.258-4.416)	0.007	2.408 (1.189-4.874)	0.015	2.335 (0.814-6.7)	0.115	
Unknown	0.850 (0.412-1.752)	0.66	1.158 (0.498-2.694)	0.733	0.627 (0.134-2.944)	0.554	

Abbreviations: OR = odds ratio, CI = confidence interval, NIH = National Institute of Health, ISCO = International Standard Classification of Occupations, UCSF = University of California, San Francisco, WHO = World Health Organization, KPS = Karnofsky Performance Scale, IDH= isocitrate dehydrogenase

Table S7. Screening nine machine learning techniques using 10-fold internal cross-validation for prediction of trial enrollment

		Machine learning technique								
		Boosted Neural Network	Boot-strap Forest	Boosted Tree	Fit Step-wise	General-ized Regress-ion Lasso	Decision Tree	Support Vector Mach-ines	Nominal Logistic	K Nearest Neigh-bors
N Trial Folds		10	10	10	10	10	10	10	10	10
Sum Freq		76.70	104.20	104.20	76.70	76.70	104.20	76.70	76.70	104.20
Validation Set Folds	RSquare	0.1757	0.0926	0.0619	0.1176	0.0947	0.0741	-0.032	0.0016	-0.046
	Mean RASE	0.42338	0.44292	0.44899	0.44405	0.44952	0.44909	0.47568	0.46063	
	Standard Deviation RASE	0.03570	0.01818	0.01190	0.03053	0.02897	0.01621	0.04189	0.03491	
	Mean AUC	0.7840	0.7136	0.6997	0.7301	0.7100	0.6730	0.6992	0.7066	
	Mean MR	0.27431	0.31098	0.32161	0.32511	0.32775	0.33122	0.33950	0.35377	0.32723
	Sensitivity	0.5399	0.3629	0.3286	0.4529	0.3659	0.1971	0.4529	0.4601	
	Specificity	0.8289	0.8540	0.8555	0.8004	0.8432	0.9075	0.7760	0.7495	
	Precision	0.7622	0.7260	0.7158	0.7224	0.7029	0.6909	0.7162	0.7118	
	Accuracy	0.7249	0.6891	0.6785	0.6754	0.6714	0.6689	0.6597	0.6454	
	F1	0.7941	0.7849	0.7795	0.7594	0.7667	0.7845	0.7449	0.7302	
	MCC	0.3849	0.2478	0.2148	0.2677	0.2378	0.1481	0.2383	0.2147	

Abbreviations: Sum Freq = Sum of Frequency Column, RASE = Root Average Squared Error, AUC = Area Under the Curve, MR = Misclassification Rate, MCC = Mathews Correlation Coefficient

Data S1. Dataset of demographic, socioeconomic, and oncologic characteristics of patients in the development and validation cohorts
